# Macrophages regulate PD-1 and CTLA-4 expression on ILC2s and their responsiveness in the tumor microenvironment

**DOI:** 10.1038/s41423-025-01347-x

**Published:** 2025-09-24

**Authors:** Cecilia Ciancaglini, Silvia Santopolo, Stefania Martini, Francesca Scordamaglia, Giuseppe Pietropaolo, Mattia Laffranchi, Giuseppe Sciumè, Guido Ferlazzo, Paola Vacca, Lorenzo Moretta, Linda Quatrini

**Affiliations:** 1 Tumor Immunology Unit, Bambino Gesù Children’s Hospital, IRCCS, Rome, Italy; 2 Innate Lymphoid Cells Unit, Bambino Gesù Children’s Hospital, IRCCS, Rome, Italy; 3 Experimental Pathology and Immunology, IRCCS Ospedale Policlinico San Martino, Genoa, Italy; 4 Pulmonology Unit, Ospedale Villa Scassi, ASL3 Genovese, Genoa, Italy; 5 Sapienza University of Rome, Rome, Italy; 6 Department of Experimental Medicine (DIMES), University of Genoa, Genoa, Italy

**Keywords:** ILC2, Immune checkpoint, Tumor microenvironment, Innate lymphoid cells, Tumour immunology

## Abstract

Chronic inflammation can induce lymphocyte dysfunction, which is characterized by the expression of inhibitory immune checkpoints. For type 2 innate lymphoid cells (ILC2s), the acquisition of a state of hyporesponsiveness associated with PD-1 expression has been reported in severe allergic inflammation. However, the regulation of ILC2 reactivity in the context of cancer is less clear. The contribution of ILC2s to the antitumor immune response depends, indeed, on the type of tumor and the distinct cellular interplay within the microenvironment. Here, we show that ILC2s in malignant pleural effusions express the immune checkpoints PD-1 and CTLA-4. An in vitro model of the ILC2‒macrophage interaction demonstrated that this crosstalk is responsible for driving CTLA-4 expression and limiting ILC2 activation. Thus, by preventing ILC2 exhaustion, macrophages maintain ILC2 responsiveness to signals from the tissue. These results reveal that, unlike PD-1 expression, CTLA-4 expression on ILC2s is associated with the maintenance of a reactive state during chronic inflammation in the tumor microenvironment.

## Introduction

ILC2s are the innate counterparts of type 2 helper T cells (Th2) and produce the type 2 cytokines IL-5, IL-13 and IL-4 in response to IL-25 and IL-33 stimulation. ILC2s are involved mainly in the innate immune response to parasites, in the development of asthma and allergic inflammation and in the repair of damaged tissues [1]. In cancer settings, the role of ILC2s is controversial. Type 2 immune responses are frequently associated with tumor progression, and an association between ILC2s and a tumor-promoting microenvironment has been shown in gastric [2], breast [3], and bladder [4] cancers and acute promyelocytic leukemia [5]. Conversely, an antitumor role for IL-33-activated ILC2s was reported in pancreatic ductal adenocarcinoma and melanoma, which is mediated by the recruitment of a dendritic cell (DC)-CD8^+^ T-cell axis and eosinophils, respectively [6, 7]. These findings demonstrate that the type of cancer and the distinct cellular interactions within the tumor microenvironment (TME) may influence the contribution of ILC2s to the antitumor immune response, leading to different outcomes.

Chronic inflammation in the TME induces a phenomenon of lymphocytic dysfunction, often referred to as exhaustion, which has been well characterized in T cells [8]. This process involves the expression of multiple immune checkpoints, which belong to the CD28 family of coinhibitory receptors and are essential for the maintenance of T-cell homeostasis and self-tolerance [9]. They include programmed cell death protein 1 (PD-1) and cytotoxic T lymphocyte-associated protein 4 (CTLA-4), whose expression has also been recently reported in ILC2s [3, 10–13].

PD-1 is not expressed on naive CD4^+^ or CD8^+^ T cells, but its expression quickly increases in a TCR-dependent manner and is associated with T-cell exhaustion [14]. PD-1 has two ligands, PD-L1 and PD-L2, which are expressed in lymphoid and nonlymphoid tissues, including tumors [15, 16]. At steady state, in mice, PD-1^+^ cells account for 20–40% of lung ILC2s and increase substantially upon influenza infection [17]. In the human setting, PD-1 is almost undetectable on the cell surface of unstimulated ILC2s, but its expression is induced during allergic inflammation [10–12] and, in particular, upon IL-33-mediated activation [6, 7, 18]. PD-1 is a critical regulator of the mature KLRG1^+^ ILC2 subset [11]^,^ and high coexpression of T-cell exhaustion markers was associated with hyporesponsive ILC2s, which were designated ‘exhausted-like ILC2s’ [19]. CTLA-4 shares its ligands CD80 and CD86 with the costimulatory receptor CD28, is upregulated on CD4^+^ and CD8^+^ T cells after initial activation and is constitutively expressed on regulatory T cells [9]. Compared with PD-1, much less is known about CTLA-4 expression and function in ILC2s. CTLA-4 expression in human ILC2s has been reported only in hepatocellular adenocarcinoma, breast cancer, and gastrointestinal cancer [3, 13].

In humans, ILC2s primarily reside at the lung mucosal barrier and are detectable in the PB. In the lung, ILC2s play key roles not only in tissue repair and homeostasis but also in the initiation of inflammation and in the cross-talk between innate and adaptive immunity [20]. As in other types of cancers, contradictory roles for ILC2s have been reported in pulmonary tumors [21, 22], with most of the data obtained using mouse models. The few studies performed in humans are often limited to the analysis of PB because of the difficulty in accessing human tissue samples. Therefore, the clear role of ILC2s and their immune checkpoints in the antitumor immune response has not yet been established in this context.

A useful tool for studying the pulmonary TME is malignant pleural effusions (mPEs), which are obtained via patient thoracentesis. mPEs contain many immune components present within solid tumors, including soluble immune mediators, lymphocytes and myeloid cells [23]. In particular, among myeloid cells, macrophages are enriched in mPE and display a transcriptional profile typical of “alternatively activated” macrophages [24]. ILC2s are able to enhance M2 macrophage polarization [25], but the reciprocal effect of macrophages on ILC2s has not been investigated thus far.

In this study, we aimed to analyze the expression and function of the immune checkpoints PD-1 and CTLA-4 on ILC2s in the context of human lung tumors. We analyzed ex vivo mPE from patients with primary and metastatic lung tumors and reproduced in vitro ILC2‒macrophage interactions to investigate the molecular mechanisms underlying immune checkpoint expression in ILC2s.

Taken together, our data provide novel insights into the biological meaning of PD-1 and CTLA-4 expression in ILC2s, revealing a novel potential role of the interplay between ILC2s and macrophages in the antitumor immune response.

## Results

### ILC2s in the lung TME express PD-1 and CTLA-4 immune checkpoints

**Fig. 1:**
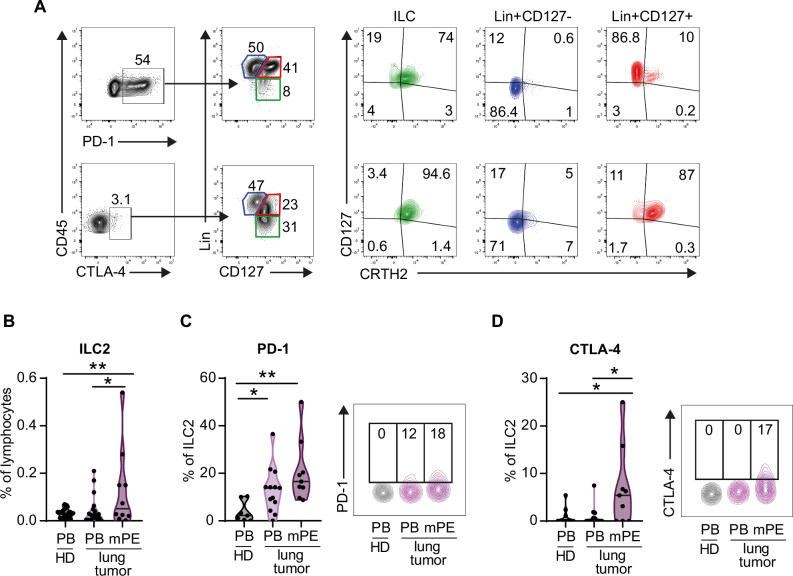
Characterization of ILC2s in lung tumor patients. **A** Proportion of CTLA-4- and PD-1-expressing CD45^+^ mononuclear cells isolated from mPE. Among PD-1^+^ or CTLA-4^+^ cells, cells were gated according to Lin (CD3, CD19, CD14, and CD56) and CD127 expression. CD127/CRTH2 expression is shown for each subset. **B** Percentages of ILC2s gated among CD45^+^ live, single cells in the lymphocyte gate drawn in the FSC/SSC plot and **C**, **D** ILC2 expression of the inhibitory checkpoints PD-1 and CTLA-4 were analyzed in HD PB, PB and mPE of patients with lung tumors. Each dot represents one donor (**B**: *n* = 22 HDs, *n *= 20 PB tumors, *n *= 10 mPE; **C** *n *= 8 HDs, *n *= 14 PB tumors, *n *= 9 mPE; **D** *n *= 10 HDs, *n *= 14 PB tumors, *n *= 9 mPE). One-way ANOVA was performed; *p < 0.05, **p < 0.01. FACS dot plots are shown for one representative lung tumor patient’s mPE and PB (**A**, **C**, **D**) and one representative HD PB sample (**C**, **D**)

To comprehensively profile the expression of the immune checkpoints PD-1 and CTLA-4 on immune cells in the lung TME, we analyzed CD45^+^ mononuclear cells isolated from malignant pleural effusions (mPEs) in patients with primary and metastatic lung tumors. Through flow cytometry analysis, we identified the cellular sources of these receptors (Fig. 1A). Compared with CTLA-4, PD-1 is expressed by a much higher proportion of cells. PD-1^+^ cells included a low fraction of ILCs [lin (CD3, CD19, CD56, CD14)^-^CD127^+^], among which ILC2s (CRTH2^+^) constituted the dominant subset (Fig. 1A). In contrast, CTLA-4^+^ cells constitute a sizable proportion of ILCs, consisting almost exclusively of ILC2s (Fig. 1A). These results reveal a previously unappreciated upregulation of PD-1 and CTLA-4 on ILC2s in mPE.

We then directly analyzed ILC2s among mPE lymphocytes and compared them to PBs from patients and healthy donors (HDs). ILC2s were identified as CD45^+^, LIN^-^, CD127^+^, and CRTH2^+^ cells (Fig. 1B and Supplementary Fig. 1A). We verified that T lymphocytes (TCR^+^ cells) did not contaminate the ILC2 gate (Supplementary Fig. 1B). We observed a significant increase in ILC2 frequency in mPE compared with HD and patients’ PB, whereas we observed no significant difference between HD and patients’ PB (Fig. 1B). We also found that the frequency of PD-1^+^ ILC2s was higher in both the PB and mPE of patients than in the PB of HD (Fig. 1C), whereas the frequency of CTLA-4^+^ ILC2s was higher in the mPE patients than in both the HD patients and patients’ PB (Fig. 1D). We did not find any correlation between age and the data shown in Fig. 1B–D for the subjects in each group, although the mean age of these patients was significantly higher than the mean age of HD patients.

These results show that ILC2s are enriched in the TME of lung cancer patients and suggest that the TME is associated with the expression of the inhibitory checkpoints PD-1 and CTLA-4.

### Macrophages in the lung TME express PD-1 and CTLA-4 ligands

**Fig. 2:**
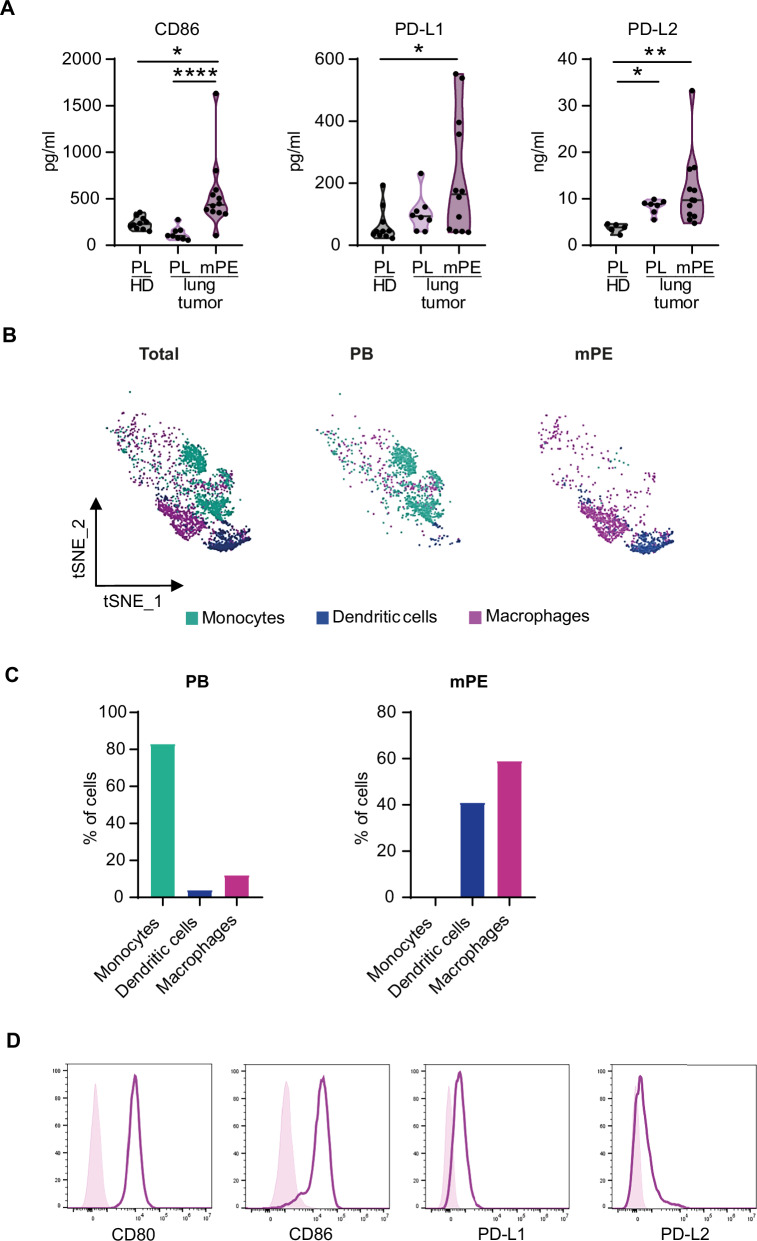
Analysis of PD-1 and CTLA-4 ligand-expressing cells in lung tumor patients. **A** Concentrations of soluble CD86, PD-L1 and PD-L2 ligands in HD PB, PB and mPE of patients with lung tumors. tSNE plots (**B**) and average proportion (**C**) within each sample type of each cell subset color-coded by subset. **D** CD80, CD86, PD-L1 and PD-L2 membrane expression on macrophages from mPE was analyzed by flow cytometry. FACS histograms are shown for one representative donor. FMO is shown as a negative control. Each dot in (**A**) represents one donor. One-way ANOVA was performed in (**A**); *p < 0.05, **p < 0.01, ****p < 0.0001

Since we observed an increase in ILC2 frequency and PD-1 and CTLA-4 expression in mPEs from lung cancer patients, we sought to characterize the soluble and cellular immune composition of the TME. To this end, we compared the concentrations of soluble analytes present in mPE to those present in patients and HD plasma (PL). The cytokines produced by “Type 2” immune cells (including ILC2s), IL-5, IL-13 and IL-4, were increased in both PLs and mPEs from lung cancer patients as compared to HD PLs (Supplementary Fig. 2A). Among the inflammatory cytokines, we observed an increase in the concentration of those that can positively and negatively regulate ILC2 function and proliferation in patients' mPE and PL. In particular, we observed that the levels of IL-33, TSLP, IL-6, TGF-β and IL-10 were increased specifically in mPE, whereas the levels of IL-2 and IL-1β were increased also in patients’ PLs compared with HD PLs (Supplementary Fig. 2B, C). Since ILC2s in the mPE express PD-1 and CTLA-4 (Fig. 1C, D), we measured the concentration of their soluble ligands in the TME. We observed that in mPE, there was a higher concentration of CD86 than in both HDs and patients’ PLs and higher concentrations of PD-L1 and PD-L2 than in HD PLs (Fig. 2A). The concentration of soluble CD80 was below the limit of detection in all the samples. To characterize the immune cell composition of mPE, we utilized a publicly available scRNA-seq dataset of mononuclear cells isolated from matched PB and mPE samples from NSCLC patients [24]. We confirmed that the major cellular compartments identified by unbiased clustering were T cells, B cells, NK cells and myeloid cells (Supplementary Fig. 3A, B). We detected *CD80*, *CD86* and *CD274* (encoding PD-L1) expression predominantly in the myeloid cluster (Supplementary Fig. 3C–E), whereas *CD273* (encoding PD-L2) was not detected, probably due to the low depth of sequencing per cell characterizing this technique. By further clustering myeloid cells, we confirmed that monocytes were enriched in PB, whereas macrophages and DCs were enriched in mPE (Fig. 2B, Supplementary Fig. 3F). Both mPE DCs and macrophages expressed *CD80*, *CD86*, and *CD274* transcripts (Supplementary Fig. 3G). Since macrophages represent the most abundant myeloid population in mPE (Fig. 2C), we focused on this cell subset to further analyze PD-1 and CTLA-4 ligand expression by flow cytometry. We detected the expression of all of these ligands on mPE macrophages, which were identified as CD14^+^CD68^+^ cells (Fig. 2D and Supplementary Fig. 1C). These data suggest that cytokines and inhibitory receptor ligands that can regulate ILC2 function are present in the lung TME. In particular, PD-1 and CTLA-4 ligands are present not only in soluble form but also on the surface of macrophages, which are enriched in mPE.

### Generation of human-activated ILC2 primary cultures

**Fig. 3:**
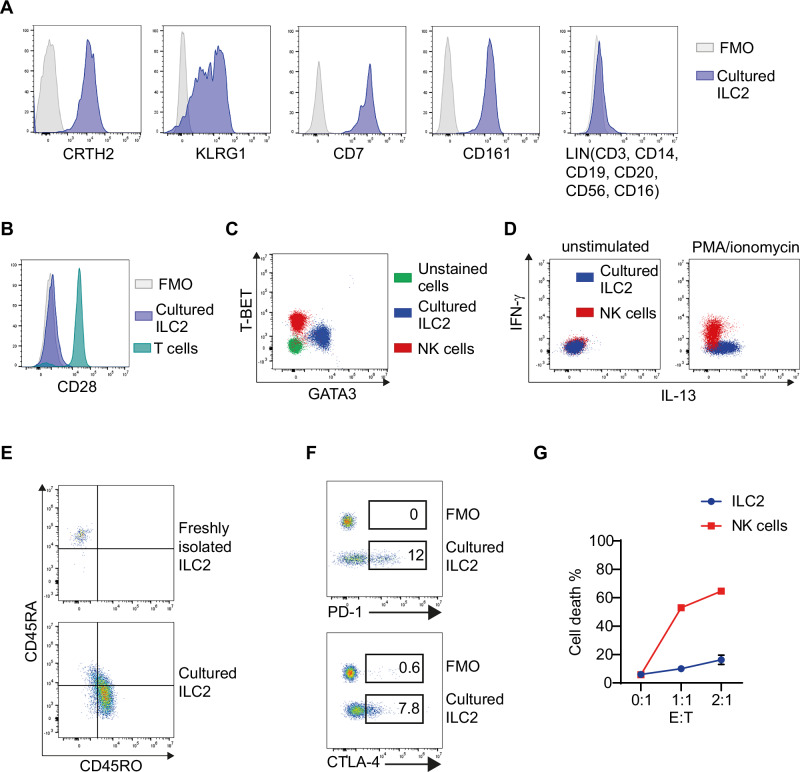
Characterization of primary ILC2 cultures. **A** Expression of CRTH2, KLRG1, CD7, CD161 and LIN (CD3, CD14, CD19, CD20, CD56, and CD16) on cultured ILC2s; **B** expression of CD28 in cultured ILC2s and T cells (gated as CD3^+^ among freshly isolated HD PBMCs); and **C** expression of GATA3 and T-BET in cultured ILC2s and cultured NK cells. **D** Intracellular IL-13 and IFN-γ staining of cultured ILC2 and NK cells unstimulated or stimulated with PMA/ionomycin. **E** Expression of CD45RA and CD45RO on freshly isolated ILC2s and cultured ILC2s. **F** PD-1 and CTLA-4 expression on cultured ILC2s. **A**, **B**, **F** FMOs are shown as negative controls. FACS plots are shown for one representative HD. **G** Percentages of dead THP-1 target cells (identified as Annexin V^+^/7-AAD^+^) after 48 h of coculture with ILC2s or NK cells at the indicated effector:target (E:T) ratios. The data are shown as the means +/−SDs of 3 independent experiments for ILC2s and 1 representative experiment for NK cells

To study in vitro the molecular mechanisms regulating immune checkpoint expression and function in ILC2s, we generated ILC2 cultures from ILC2s freshly isolated from HD PBMCs. Unlike other published protocols, we did not flow-sort ILC2s [26–28] or expand them with feeder cells [26, 28]. We established a new protocol to generate in two weeks primary ILC2 cultures, consisting of ILC2 enrichment, expansion/activation with cytokines (IL-2, IL-7 and IL-1β) [17] and negative selection by non-ILC2 depletion. ILC2 cultures that we generated in vitro were characterized phenotypically and functionally. They were >95% pure; expressed the markers CRTH2, KLRG1, CD7, and CD161 and lacked the expression of CD3, CD14, CD19, CD20, CD56, CD16 and CD28 (Fig. 3A, B). Moreover, this population expressed the transcription factor GATA3, very low levels of T-BET (Fig. 3C) and, accordingly, produced IL-13 but not IFN-γ upon PMA/ionomycin stimulation (Fig. 3D). We observed that the expression of CD45RO increased, whereas the expression of CD45RA decreased on ILC2s after two weeks in culture compared with freshly isolated ILC2s, which uniformly expressed CD45RA (Fig. 3E). We also found that PD-1 and CTLA-4 expression on cultured ILC2s was upregulated (Fig. 3F) compared with that on freshly isolated ILC2s (Fig. 1C, D). Moreover, in agreement with the findings of Li et al. [26], cultured ILC2s displayed weak cytotoxic activity against a tumor cell line (Fig. 3G).

All these data demonstrate that we generated in vitro-activated primary ILC2 cultures that express typical markers of this cell lineage and the inhibitory checkpoints PD-1 and CTLA-4, thus displaying a phenotype similar to that of ILC2s from mPE (Fig. 1C, D).

Given that both ILC2s and macrophages are present in the lung TME and express the inhibitory receptors PD-1 and CTLA-4 and their ligands, respectively, to study the crosstalk between ILC2s and macrophages in the TME, we performed in vitro coculture experiments between primary cultured ILC2s and monocyte-derived macrophages (MoMs obtained from HD PBMCs). MoMs naturally expressed the PD-1 and CTLA-4 ligands CD80, CD86, PD-L1 and PD-L2 on the cell surface, similar to macrophages from mPE (Supplementary Fig. 4A). In addition, MoMs expressed the typical markers of the M2 phenotype, CD163 and CD206, and the latter was upregulated upon coculture with ILC2s (Supplementary Fig. 4B). Compared with MoMs cultured alone, coculture-derived MoMs (sorted as shown in Supplementary Fig. 5A) also released lower amounts of the inflammatory cytokines IL-1β, IL-6 and TNF in response to LPS stimulation (Supplementary Fig. 4C).

These data show that in our coculture system, ILC2s promote M0 polarization toward M2 and that the ILC2-MoM coculture system represents a simplified model to reproduce this in vitro cellular crosstalk in the TME.

### Coculture with MoM limits ILC2 activation

**Fig. 4:**
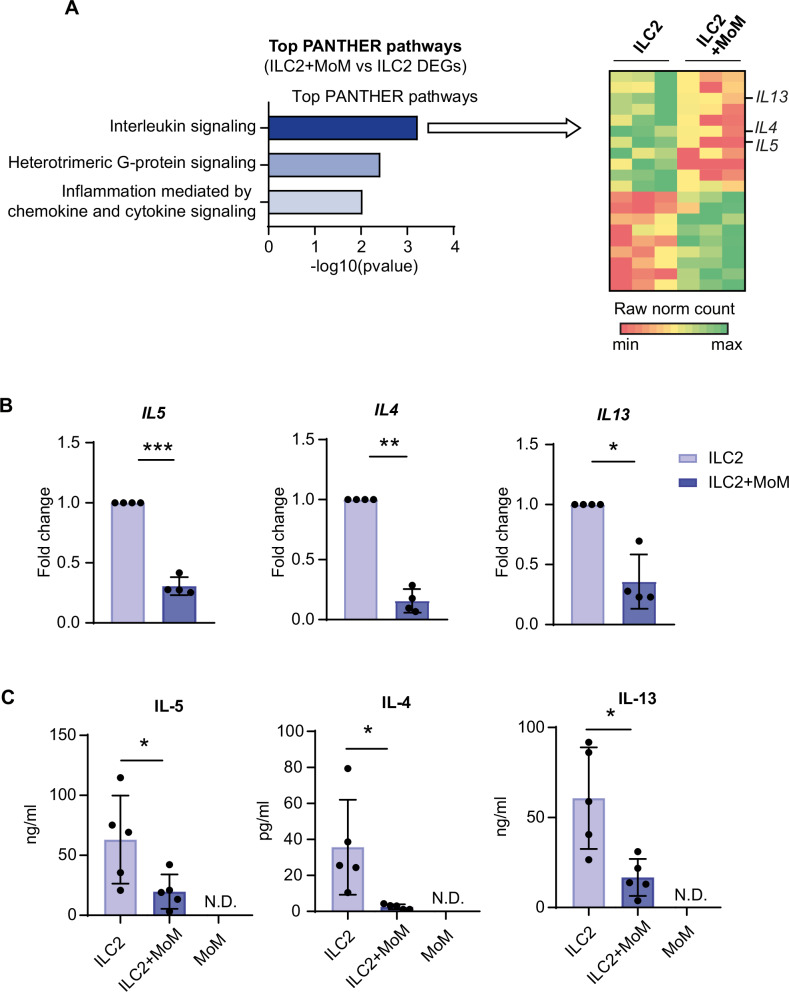
Macrophages inhibit ILC2 effector functions. **A** RNA sequencing was performed to compare gene expression between ILC2s sorted from coculture with MoMs and ILC2s cultured alone. DEGs between each pair of samples were identified from the normalized gene-level counts via the following cutoff values: fold change of 2 and adjusted *P* value ≤ 0.05. Pathway enrichment analysis was performed with Panther on the set of DEGs between ILC2s cocultured with MoMs and ILC2s cultured alone. The heatmap shows normalized counts for the DEGs belonging to the “Interleukin signaling” pathway in the 2 conditions (*n *= 3 donors). **B** *IL5*, *IL4* and *IL13* gene expression normalized to that of *18S* was confirmed by RT‒PCR. The histograms show the fold change relative to ILC2s. **C** IL-5, IL-4 and IL-13 cytokine concentrations were also analyzed in the supernatant after coculture by bead-based multiplex flow cytometry. N.D. not detected. The histograms show the means +/− SDs of 4 (**B**) and 5 (**C**) independent experiments. Ratio paired *t* tests between 2^−ΔCt^ values (**B**) and t tests (**C**) were performed. *p < 0.05, **p < 0.01, ***p < 0.005

ILC2s and MoMs were cocultured for 4 days in medium containing IL-2 and IL-7. We focused our analysis on the effect of MoM on ILC2s by performing RNA sequencing to compare gene expression between ILC2s sorted from coculture and ILC2s cultured alone (Supplementary Fig. 5B). Pathway enrichment analysis revealed that the (Differentially expressed genes) DEGs were involved mainly in the interleukin signaling pathway, including genes encoding the type 2 cytokines IL-5, IL-4 and IL-13, whose expression was downregulated in ILC2s upon coculture with MoMs (Fig. 4A). We confirmed this finding directly at the transcript level by RT‒PCR (Fig. 4B) and at the protein level by analyzing the cytokines released into the supernatant during coculture (Fig. 4C). We found a significant decrease in the expression and release of IL-5, IL-4 and IL-13 by ILC2s cocultured with MoMs compared with ILC2s cultured alone.

Taken together, these results demonstrate that macrophages inhibit ILC2 effector functions during persistent cytokine stimulation.

### PD-1 and CTLA-4 ligands are able to suppress ILC2 effector functions

**Fig. 5:**
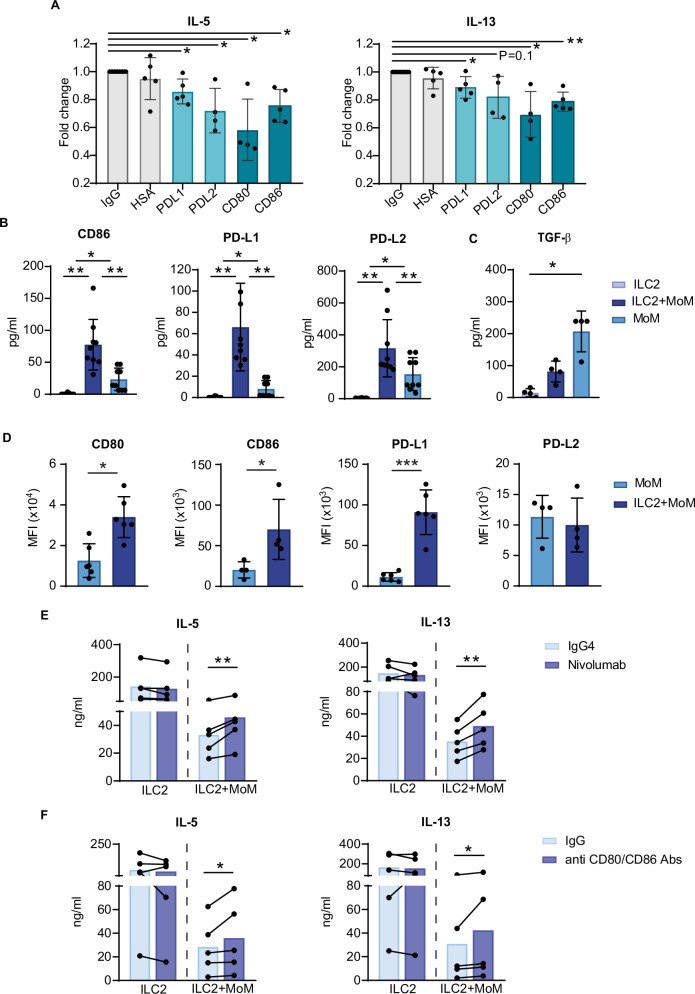
Inhibitory effects of PD-1 and CTLA-4 on ILC2s. **A** Concentrations of IL-5 and IL-13 released into supernatants by ILC2s stimulated with IL-2 for 24 h in the presence of plate-bound PD-L1, PD-L2, CD80 and CD86 recombinant Fc chimeric proteins; IgG-Fc and HSA were used as controls. The histograms show the fold change relative to that of IgG. **B** CD86, PD-L1, PD-L2 and **C** TGF-β concentrations in the supernatants and **D** CD80, CD86, PD-L1 and PD-L2 surface expression on MoMs after ILC2–MoM coculture. The histograms show the means +/− SDs of at least 4 (**A**–**D**) independent experiments. **E**, **F** IL-5 and IL-13 concentrations in the supernatants of cocultures performed in the presence of antibodies blocking PD-1 (nivolumab) or CD80 and CD86 (anti-CD80/CD86 Abs). The histograms show the means of 5 independent experiments; lines connect paired samples. A *t* test was performed in (**D**), and a ratio paired t test was performed in (**A**), (**E**) and (**F**). An one-way ANOVA test was performed in (**B**) and (**C**). *p < 0.05, **p < 0.01, ***p < 0.005

To understand the molecular mechanisms by which MoMs downregulate ILC2 effector functions, we examined the contribution of immune checkpoint receptor‒ligand interactions. First, we directly investigated whether these molecules are able to negatively regulate ILC2 function and the downstream targets. To this end, primary cultured ILC2s were stimulated with IL-2 for 24 h in the presence of recombinant PDL-1, PDL-2, CD80 or CD86. After stimulation, we analyzed the concentrations of IL-5 and IL-13, whose expression is induced by IL-2 in ILC2s, in the supernatants. We found that cytokine release decreased in the presence of each ligand compared with that of the Ig control, with CD80 having the greatest inhibitory effect (Fig. 5A). These results demonstrate that PD-1 or CTLA-4 triggering of ILC2s negatively affects their effector functions.

We next investigated whether PD-1 and CTLA-4 ligands were also released into the supernatant upon coculture. Our results revealed that compared with MoM culture alone, coculture with ILC2s increased the release of soluble CD86, PD-L1 and PD-L2 (Fig. 5B). Since TGF-β has been demonstrated to have an inhibitory effect on ILC2s [29], we also analyzed the TGF-β concentration. We observed that, in contrast with immune checkpoint soluble ligands, MoMs released a lower amount of TGF-β in the supernatant upon coculture than MoMs cultured alone did (Fig. 5C). CD80, CD86 and PD-L1, but not PD-L2, surface expression was also upregulated on MoMs upon coculture (Fig. 5D).

To determine the contribution of the PD-1 and CTLA-4 pathways to the inhibition of ILC2 cytokine production mediated by interactions with MoM, we performed coculture experiments in the presence of antibodies (Abs) blocking PD-1 (nivolumab) or CD80 and CD86 (anti-CD80/CD86 Abs). We observed a modest, although significant, effect of blockade of these pathways on the secretion of IL-5 and IL-13 by ILC2s (Fig. 5E, F).

These data demonstrate that MoMs express and release PD-1 and CTLA-4 ligands during coculture with ILC2s and that these inhibitory pathways contribute to the MoM-dependent suppression of ILC2 effector functions.

### Coculture with MoM modifies PD-1 and CTLA-4 expression on ILC2s

**Fig. 6:**
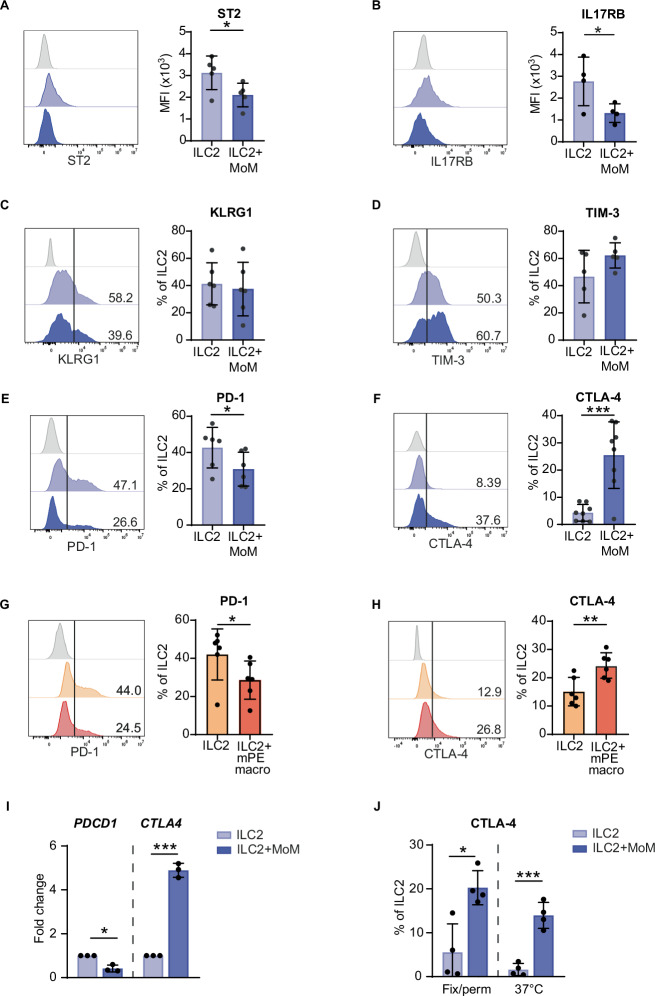
PD-1 and CTLA-4 are differentially regulated on ILC2s by MoM. **A**–**F** ILC2 expression of ST2, IL-17RB, KLRG1, TIM-3, PD-1 and CTLA-4 after coculture with MoMs. **G**, **H** ILC2 expression of PD-1 and CTLA-4 after coculture with mPE macrophages. **I ***PDCD1* and *CTLA4* gene expression was normalized to that of *GAPDH* by RT‒PCR. The histograms show the fold change relative to ILC2s. **J** CTLA-4 expression on ILC2s upon coculture was detected by staining cells after fixation and permeabilization or by staining live cells at 37 °C. In (**A**–**H**), the FACS histograms on the left show one representative experiment. FMO is shown in gray as a negative control. The histograms show the means +/− SDs of at least 4 independent experiments. A *t* test was performed (**A**–**H**, **J**), and a ratio paired t test was performed to compare the 2^−ΔCt^ values (**I**); *p < 0.05, **p < 0.005, ***p < 0.001

To verify whether coculture with MoMs could modify the ILC2 phenotype in parallel with ILC2 function, we assessed the expression of typical receptors and inhibitory checkpoints on ILC2s upon coculture. The expression of the costimulatory receptor ICOS was not significantly affected (Supplementary Fig. 6A), whereas the expression of the IL-33 receptor ST2 and the IL-25 receptor IL17RB (but not that of IL17RA, Supplementary Fig. 6B) was downregulated in ILC2s upon coculture with MoM (Fig. 6A, B). TIGIT expression was not detected in ILC2s (Supplementary Fig. 6C). We did not observe any difference in the expression of KLRG1 or TIM-3 between ILC2s cultured alone and those cultured with MoM (Fig. 6C, D). Instead, the percentage of PD-1^+^ ILC2s was lower, whereas the percentage of CTLA-4^+^ ILC2s was higher in ILC2s cultured with MoM than in ILC2s cultured alone (Fig. 6E, F). Similar results were obtained by coculturing ILC2s with macrophages generated from the PB monocytes of lung tumor patients (MoM Tum) (Supplementary Fig. 6D–F) and with macrophages directly isolated from mPE (mPE macro) (Fig. 6G, H and Supplementary Fig. 6G).

Next, we focused on PD-1 and CTLA-4 and observed that the modifications in protein amount were accompanied by parallel modifications in transcript abundance (Fig. 6I). The CTLA-4 protein is rapidly internalized to create a majority of the intracellular pool at steady state in T cells [30]. To verify whether the increase in total CTLA-4 protein levels observed upon staining fixed and permeabilized cells (Fig. 6F) corresponded to an increase in the amount of protein expressed on the cell surface, we performed staining on live cells at 37 °C to allow CTLA-4 recycling (Fig. 6J). Our data show that coculture with MoM induced an upregulation of CTLA-4 expression on the ILC2 cell membrane, increasing the amount of receptor available for ligand binding.

Collectively, these results demonstrate that macrophages differentially modulate the expression of PD-1 and CTLA-4 on ILC2s.

### CTLA-4 upregulation in ILC2s is inhibited by CD80/CD86 blockade and is reversible

**Fig. 7:**
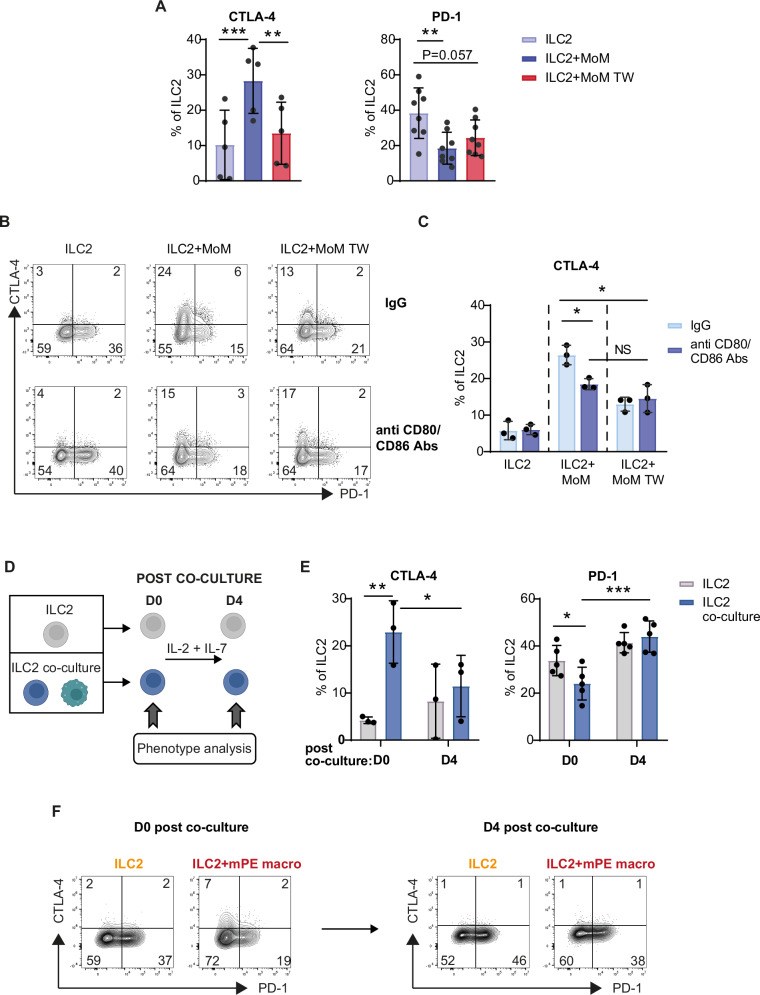
CTLA-4 upregulation on ILC2s is mediated by its ligands. **A** PD-1 and CTLA-4 expression on ILC2s after coculture with MoM with or without TW. **B**, **C** Representative FACS dot plots and histograms showing PD-1 and CTLA-4 expression on ILC2s after coculture in the presence of anti-CD80 and anti-CD86 Abs. **D** Experimental design (created in BioRender). CTLA-4 and PD-1 expression on ILC2s after coculture with MoM (**E**) or mPE macro (**F**) (D0) or 4 days post-culture (D4). The histograms show the percentage of positive cells expressed as the mean +/− SD of at least 5 (**A**) and 3 (**C**, **E**) independent experiments. FACS plots from one representative experiment out of 2 performed are shown in (**F**). One-way ANOVA was performed; NS not significant. *p < 0.05, **p < 0.01, ***p < 0.001

To understand the mechanism underlying the differential regulation of PD-1 and CTLA-4 expression, we performed ILC2–MoM coculture experiments in the presence of a transwell (TW) to investigate the contribution of cell‒cell contact. We found that the increase in CTLA-4^+^ ILC2s induced by coculture with MoM was inhibited by the presence of TW. Conversely, the percentage of PD-1^+^ ILC2s was lower in cocultures with MoM than in those with ILC2s cultured alone, regardless of the presence of TW (Fig. 7A). These data demonstrate that the regulation of these receptors on ILC2s is mediated by distinct mechanisms: PD-1 expression is controlled mainly by soluble factors, whereas CTLA-4 expression requires cell-to-cell contact. To investigate the molecular mechanisms through which ILC2–MoM cell contact induces CTLA-4 upregulation in ILC2s, we performed coculture experiments in the presence of anti-CD80 and anti-CD86 antibodies to block CTLA-4 ligands (Fig. 7B, C). We found that the expression of CTLA-4 in ILC2s cultured alone was similar between the control (Ig) and anti-CD80/CD86 Abs conditions. Instead, the percentage of CTLA-4^+^ ILC2s in cocultures performed in the presence of anti-CD80/CD86 Abs was lower than that in the coculture control conditions. In TW cocultures, the percentage of CTLA-4^+^ ILC2s was similar between the control and anti-CD80/CD86 Abs conditions (Fig. 7B, C). Therefore, when CTLA-4 ligands are blocked, CTLA-4 upregulation in ILC2s is inhibited. Interestingly, upon CD80/CD86 blockade, the expression of CTLA-4 was similar between ILC2s cocultured with and without TW, suggesting that CTLA-4 upregulation induced by cell‒cell contact is completely dependent on its ligands (Fig. 7B, C). Importantly, PD-1 expression on ILC2s was not affected by CD80/CD86 blockade (Fig. 7B, C). Moreover, PD-1/CTLA-4 costaining revealed mutually exclusive expression of these receptors by ILC2s, confirming the expression pattern observed in mPE ILC2s (Supplementary Fig. 1A). These results demonstrate that ILC2–MoM cell contact mediated by CD80 and CD86 is required to induce CTLA-4 upregulation in ILC2s.

We next analyzed the modifications in the expression levels of PD-1 and CTLA-4 immune checkpoints on ILC2s after coculture (Fig. 7D). In these experiments, we cocultured ILC2s with either MoM or mPE (mPE macro. Postcoculture (D0) phenotype analysis confirmed the differential effects of both MoM (Fig. 7E) and mPE macro (Fig. 7F, left panel) on ILC2 PD-1 and CTLA-4 expression. ILC2s were sorted postculture and cultured again in the presence of IL-2 and IL-7 (Fig. 7D). The percentage of ILC2s cultured alone that expressed CTLA-4 and PD-1 did not change significantly when the cells were restimulated for 4 days (D4) (Fig. 7E, F right panel). Instead, we observed that the percentage of ILC2s that expressed CTLA-4 significantly decreased at D4 compared with that of ILC2s analyzed postcoculture with either MoM or mPE macro (D0), whereas the percentage of PD-1-expressing cells significantly increased under the same conditions (Fig. 7E, F right panel).

These results demonstrate that CTLA-4 upregulation in ILC2s upon coculture with macrophages is a reversible process.

### Macrophages maintain ILC2 responsiveness to environmental stimuli

**Fig. 8:**
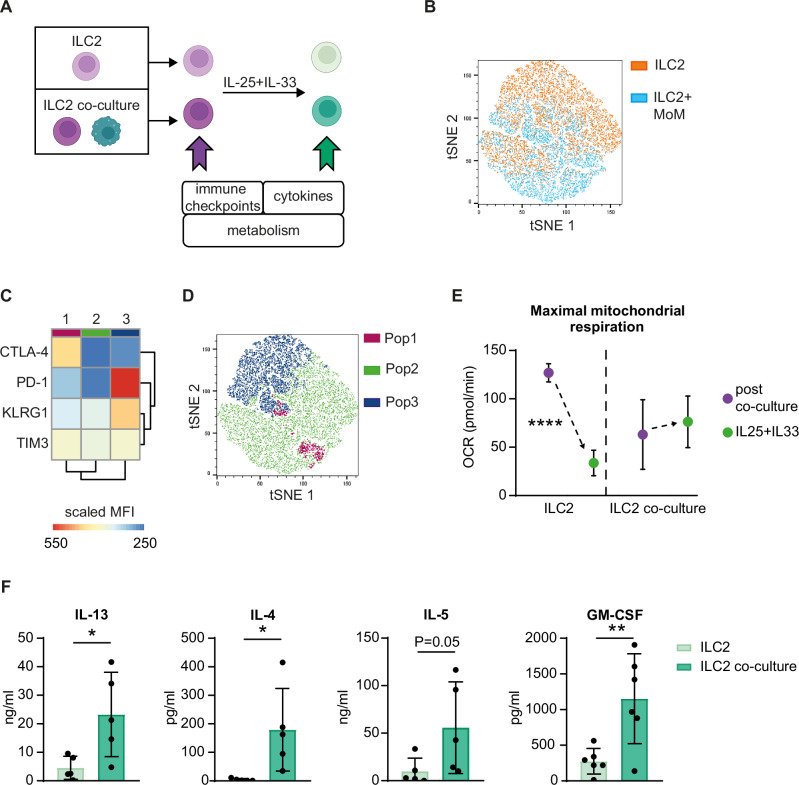
Macrophages prevent ILC2 exhaustion. **A** Experimental design (created in BioRender): ILC2s were cultured alone (ILC2s) or cocultured with MoM (ILC2 coculture), and the indicated analyses were performed immediately and/or after stimulation with IL-25 + IL-33. **B**–**D** tSNE (t-distributed stochastic neighbor embedding) and FlowSOM were run on concatenated ILC2 and ILC2+MoM fcs files on the basis of CTLA-4, PD-1, KLRG1 and TIM-3 expression analyzed by flow cytometry after coculture. T-SNE overlays with samples (**B**) and with the populations identified by FlowSOM (**D**), and the FlowSOM heatmap (**C**) are shown. The scale bar in (**C**) indicates the scaled mean fluorescent intensity (MFI) from the minimum (blue) to the maximum (red). **E** Seahorse analysis of the oxygen consumption rate (OCR) was performed on ILC2s immediately after coculture (purple dots) and after stimulation with IL-25 and IL-33 (green dots). **F** Concentrations of cytokines released by ILC2s stimulated with IL-25 and IL-33 for 3 days in the supernatant after coculture. The data are shown as the means +/− SDs of 2 (**E**) and at least 5 (**F**) independent experiments. A *t* test was performed; *p < 0.05, **p < 0.01, ****p < 0.001

The persistent expression of an immune checkpoint marks “exhausted” lymphocytes, which are usually characterized by the coexpression of other immune checkpoints [31], a quiescent metabolic status, and a lower responsiveness to activating signals [32]. We tested these 3 features of “exhaustion” in ILC2s after coculture with macrophages (Fig. 8A). To visualize the coexpression of immune checkpoints on ILC2s at the single-cell level, we performed t-distributed stochastic neighbor embedding (t-SNE) analysis on concatenated ILC2 samples postculture with (light blue) and without (orange) MoM (Fig. 8B) and stained them with antibodies against CTLA-4, PD-1, KLRG1 and TIM3. On the basis of the expression levels of these markers, the cells were clustered in an unsupervised manner into 3 populations (Fig. 8C, D): Pop2 included ILC2s expressing low levels of each immune checkpoint; Pop1 included ILC2s expressing the highest level of CTLA-4; and Pop 3 included ILC2s coexpressing PD-1, KLRG1 and TIM3 but not CTLA-4. While Pop1 and 2 were enriched in the ILC2+MoM sample, Pop3 was enriched in the ILC2 sample (Fig. 8B, D). These data suggest divergent expression pathways for CTLA-4 compared with the other immune checkpoints, with the former upregulated in ILC2s cocultured with MoM and the latter (PD-1, KLRG1 and TIM3) upregulated in ILC2s cultured alone. The opposite effects of PD-1 and CTLA-4 on ILC2s upon coculture with macrophages, together with the coexpression of other immune checkpoints with PD-1 only, suggest that the upregulation of these two receptors may be associated with differential ILC2 reactivity to tissue-derived signals. To verify this hypothesis, ILC2s were sorted from MoMs and cultured again in the presence of the alarmins IL-25 and IL-33 (Fig. 8A). The metabolic status of ILC2s was assessed via extracellular flux analysis of the oxygen consumption rate (OCR) after coculture and post-alarmin stimulation (Fig. 8E). OCR analysis of maximal mitochondrial respiration revealed that the increased activation status of ILC2s cultured alone was accompanied by high metabolic activity after coculture, but the mitochondrial respiratory capacity of ILC2s decreased upon further stimulation with tissue-derived signals (Fig. 8E and Supplementary Fig. 7A). Conversely, mirroring MoM-induced inhibition, coculture-derived ILC2s displayed moderate metabolism upon coculture but maintained efficient mitochondrial respiratory capacity upon IL-25 and IL-33 stimulation (Fig. 8E and Supplementary Fig. 7B). Moreover, coculture-derived ILC2s had a greater ability to secrete IL-13, IL-4, IL-5 and GM-CSF in response to IL-25 and IL-33 stimulation than did ILC2s cultured alone (Fig. 8F), which was accompanied by the upregulation of ST2 (Supplementary Fig. 7C). Increased IL-4 and IL-5 production in response to alarmin stimulation was confirmed in ILC2 cultured with MoM Tum and mPE macro compared to ILC2 cultured alone (Supplementary Fig. 7D).

In contrast, coculture with MoMs had no effect on ILC2 cytotoxicity (Supplementary Fig. 7E).

Collectively, the decreased coexpression of immune checkpoints, together with the more responsive metabolic status and the increased type 2 cytokine production of coculture-derived ILC2s, demonstrate that, by limiting ILC2 activation, macrophages prevent their exhaustion, thus rendering ILC2s more reactive to tissue-derived signals.

## Discussion

In the present study, we provide insight into the interplay between ILC2s and macrophages in the antitumor immune response. Our data highlight the differential effects of PD-1 and CTLA-4 expression on ILC2s, which are associated with distinct molecular mechanisms and responsivity to tissue-derived signals.

We demonstrated that ILC2s are present in the lung TME and that they express PD-1, which is not expressed on ILC2s in HD-PB. These data are in line with other studies that reported an increase in tumor-infiltrating ILC2s in NSCLC and that tumor-associated ILC2s express PD-1 [25, 33]. Moreover, we demonstrated for the first time that ILC2s expressed CTLA-4 in mPE and that, unlike PD-1, its expression was specific to the TME, as it was not observed in circulating ILC2s from lung tumor patients.

A major limitation in the study of ILC2s, particularly in humans, is their low frequency in PB and tissues. To overcome this limitation, we set up conditions to generate primary ILC2 cultures from HD PB. We obtained a relatively high number of activated ILC2s in culture to be able to perform mechanistic experiments in vitro, and the tool we developed will also be useful in the future to investigate other aspects of ILC2 biology. ILC2s in culture expressed CD45RO, a marker of activated ILC2s, similar to “inflammatory” ILC2s in mice, while freshly isolated ILC2s uniformly expressed CD45RA, a marker of resting ILC2s [34]. Moreover, activated ILC2s in culture expressed the inhibitory checkpoints PD-1 and CTLA-4, thus displaying a phenotype similar to that of ILC2s from mPE. To investigate the molecular mechanisms that control ILC2 immune checkpoint expression and function in the TME, we generated a simplified model of the TME in vitro, with a focus on ILC2–macrophage crosstalk. We confirmed that macrophages were enriched in mPE [24] and demonstrated that they expressed PD-L1, PD-L2, CD80 and CD86. As a surrogate for mPE macrophages, we used monocyte-derived macrophages from HDs, which express similar markers, including PD-1 and CTLA-4 ligands, and displayed M2 polarization upon coculture with ILC2s, as previously reported [25].

By performing coculture experiments, we demonstrated that MoM inhibited ILC2 activation. Using recombinant PD-1 and CTLA-4 ligands, we showed that, in principle, they are able to reduce ILC2 release of type 2 cytokines, and we also observed that these immune checkpoint pathways play a partial role in MoM-mediated ILC2 inhibition. The other MoM-derived factors contributing to the limitation of ILC2 effector functions remain to be determined. We hypothesize that TGF-β may play a role since it was previously shown that TGF-β suppresses ILC2 activation in humans [29]^,^ and we detected this cytokine in the coculture supernatant. Our data indicate that, unlike T cells, in which PD-1 and CTLA-4 directly antagonize TCR-activating signals at immunological synapses [35, 36], these immune checkpoints have a weak inhibitory capacity in ILC2s. Nevertheless, we observed that, similar to that on T cells, PD-1 expression on ILC2s matches the level of cell activation: along with their functional impairment, coculture-derived ILC2s showed lower PD-1 expression than did ILC2s cultured alone, while PD-1 expression was upregulated upon separation from MoMs and further activation. Chronically activated ILC2s that were not cocultured with macrophages coexpressed PD-1 with the immune checkpoints KLRG1 and TIM3 and became hyporesponsive upon further restimulation. Our data are in line with the definition of PD-1 as a marker of “exhausted like” ILC2s [19]. Indeed, in mice, activated ILC2s enter an exhausted state in both severe allergic inflammation and lung melanoma metastasis, characterized by increased coexpression of inhibitory receptors and reduced metabolism and responsiveness to cytokines [12, 37].

Interestingly, we observed opposite CTLA-4 and PD-1 regulation in ILC2s upon coculture with MoMs, which was also confirmed in cocultures performed with patient-derived macrophages. This opposite regulation was detected at both the transcript and protein levels, suggesting that the expression of these 2 receptors has different implications in the ILC2 immune response. This may also explain why PD-1 and CTLA-4 were not coexpressed on ILC2s but were alternatively expressed by different subsets. While PD-1 expression was decreased by coculture with MoM, CTLA-4 expression was increased. This differential regulation is mediated by distinct molecular mechanisms: decreased PD-1 expression is dependent mainly on the inhibition of cell activation by soluble factors released by MoMs, whereas CTLA-4 upregulation requires cell-to-cell contact mediated by its ligands. Unlike other immune checkpoints, CTLA-4 has one of the shortest known half-lives among transmembrane proteins [38, 39]. In T cells, most CTLA-4 molecules are found within intracellular compartments, are constantly cycling to the cell surface [40] and are rapidly turned over by lysosomal degradation [41]. Interaction with CD80 and/or CD86 likely induces CTLA-4 expression at the transcriptional level and/or reduces its degradation. Ligand-dependent upregulation of CTLA-4 expression on ILC2s represents a novel regulatory mechanism since, until recently, only cytokine cues were identified as stimuli able to induce this receptor on ILCs [42, 43]. Moreover, we observed that, unlike PD-1, CTLA-4 upregulation on ILC2s is reversible and uncoupled from the expression of other inhibitory checkpoints. Together, this evidence leads to the hypothesis that CTLA-4 is not a marker of exhaustion for ILC2s, unlike PD-1. We hypothesize that CTLA-4-mediated inhibition is a temporary “brake” induced by MoM and that CTLA-4 on ILC2s serves to increase the threshold for cell activation to induce productive signaling, similar to what was hypothesized for T cells [44]. In line with this, we showed that, by limiting ILC2 activation during coculture, MoMs prevent ILC2 exhaustion and preserve their responsiveness to tissue-derived signals. Indeed, in response to IL-25 and IL-33 stimulation, ILC2s cultured in the absence of MoM displayed a defective release of type 2 cytokines together with a reduced metabolic status, which have both been demonstrated as features of exhausted T cells [32, 45]. In line with the role of CTLA-4 suggested for ILC2s in the lung, an association between CTLA-4 upregulation and the maintenance of a reactive state has been demonstrated for ILCs in the intestine [42]. Here, immune reactions are continuously elicited by the commensal microbiota, and CTLA-4^+^ ILCs maintain their reactivity and restrain inflammation. Importantly, ILC2s isolated from cocultures presented reduced expression of the receptors IL-33 and IL-25, demonstrating that the differential responsiveness to these cytokines was dependent on the cell status rather than on the number of receptors available in our experimental model.

The general consequences of the overall immune response in the TME of the macrophage-mediated regulation of ILC2 responsiveness remain to be determined. We propose a model in which ILC2s and macrophages reciprocally promote each other’s protumoral functions, with ILC2s inducing M2 polarization and macrophages preserving the ability of ILC2s to respond to tissue-derived alarmins by preventing their exhaustion. As a result, this interaction may further increase growth factor production in the TME and angiogenesis and promote tumor proliferation and metastasis.

In conclusion, we provide the first evidence that CTLA-4 is expressed on ILC2s in the lung TME. Although we cannot exclude the contribution of many other components of the TME, our data strongly suggest that ILC2‒macrophage crosstalk is one of the main mechanisms responsible for CTLA-4 expression and maintenance of ILC2 responsiveness to tissue-derived signals.

## Materials and methods

### Study patients and sample collection

Blood samples and mPEs following thoracentesis were obtained from patients who were admitted to Villa Scassi Hospital (Genoa, Italy) following the approval of the Azienda Sanitaria Locale 3 Ethics Board (ID 33533184). We collected these samples from 50 patients (mean age 73, 71% male) who received a diagnosis of mesothelioma (28%), adenocarcinoma (34.5%), squamous cell lung carcinoma (9.4%), small cell lung carcinoma (9.4%) or metastatic tumors (18.7%). Blood and mPE samples were centrifuged at 800 × *g* for 10 min to obtain plasma and mPE supernatants, respectively. Subsequently, mononuclear cells (MCs) from PB and mPE were isolated via density gradient separation with Ficoll–Hypaque (Lympholyte-H, Cederlane) and used for phenotypic analysis via flow cytometry.

The PB of HD from buffy coats was used as a control (mean age 44, 80% males). Buffy coats were collected from volunteer blood donors admitted to the blood transfusion service of IRCCS Bambino Gesù Children’s Hospital (Rome, Italy) after providing informed consent, following the approval of the hospital’s Ethics Board (ID AIRC5x1000 #21147). The study was conducted following the ethical principles stated in the Declaration of Helsinki.

### Flow cytometry analysis and antibodies

For flow cytometry analysis, the cells were first stained with a LIVE/DEAD Fixable Blue Dead Cell Stain Kit (Invitrogen, Thermo Fisher Scientific) and surface antibodies in PBS supplemented with 5% FCS for 20 min at 4 °C. For intranuclear transcription factor staining, the cells were fixed, permeabilized, and stained with the FOXP3/Transcription Factor Staining Buffer Kit (Miltenyi). For cytokine and CTLA-4 staining, the cells were fixed, permeabilized, and stained with BD Cytofix/Cytoperm Plus (BD Biosciences). For CTLA-4 membrane staining, the cells were incubated for 1 h at 37 °C with an anti-CTLA4 antibody, and the remaining antibodies were added for 20 min at 4 °C. The following antibodies were used: lineage cocktail 1 [CD3 (clone SK7), CD14 (clone MφP9), CD19 (clone SJ25C1), CD20 (clone L27), CD56 (clone NCAM16.2), CD16 (clone 3G8)]-FITC, CD3- BV786 (clone HIT3α), CD14-BV786 (clone M5E2), CD19-BV786 (clone HIB19), CD279 (PD-1)-PE (clone PD-1.3.1.3), PD-1-BV650 (clone EH12), TIM-3-BV421 (clone 7D3), CD7-(PE-CF594) (clone M-T70), PDL1-(PE-CF594) (clone MIH1), IL-17RA-PE (clone W23-251), TCRαβ-BV786 (clone T10B9.1A-31) purchased from BD, CD45-(APC-AF750) (clone J33), CD45-ECD (clone J33) purchased from Beckman, CD127-BV421 (clone A019D5), IL5-PE (clone JES1-39D10), CD45RA-(APC-Cy7) (clone HI100), CD45RO-BV421 (clone UCHL1), KLRG1-FITC (clone SA231A2), TIGIT-PE/Dazzle 594 (clone A15153G) purchased from Biolegend, GATA3-PE (clone TWAJ), T-bet-(PC7) (clone 4B10), IFN-γ-(APC-eFluor780) (clone 4S. B3) purchased from eBiosciences, CD294 (CRTH2)-PE (clone BM16), KLRG1-APC (clone REA261), CTLA-4-APC (clone BNI3), CD127-APC (clone REAL102), CD80-PE (clone REA661), CD86-APC (clone REA968), PDL2-(PE-Vio615) (clone REA985), CD161-PE (clone 191B8), IL13-PE (clone JES10-5A2.2), IL4-APC (clone REA895), CD163-(PerCP-Vio700) (clone REA812), CD206-VioBlue (clone DCN228), CD278 (ICOS)-FITC (clone REA192), and CD28-VioBright FITC (clone REA612) purchased from Miltenyi, and ST2-AlexaFluor488 (clone 2154B), ST2-APC (Polyclonal Goat IgG), and IL-17RB-PE (clone 170220) purchased from R&D. Samples were acquired on a CytoFLEX LX (Beckman Coulter) by CytExpert Software v2.5.0.77 (Beckman Coulter). Flow cytometry data were analyzed with FlowJo version 10.8.1 (BD).

To cluster ILC2 subsets according to immune checkpoint expression, fcs files corresponding to stained ILC2 samples (ILC2s cultured alone and with MoM from the same donor) were concatenated using the same number of events for each condition. FlowJo tSNE and FlowSOM [46] were run on the concatenated file: FlowSOM generated the heatmap for each channel used, and each FlowSOM population (e.g., Pop1) identified was overlaid on the tSNE plot.

### ILC2 isolation and culture

ILC2s were enriched from HD PBMCs via negative selection via the RosetteSep Human ILC2 Kit (StemCell Technologies) and cultured with CTS NK-Xpander basal medium (Gibco) supplemented with 10% FBS (Gibco), glutaMAX, penicillin‒streptomycin, IL-2 (R&D), IL-7 and IL-1β (Miltenyi) (50 ng/ml each) for 2 weeks. After ILC2 enrichment from human HD PBMCs, the total cell number typically falls within the range of 1–3 × 10^6^ cells. Four days before coculture, ILC2s were further isolated via negative selection via magnetic beads with an antibody cocktail recognizing lineage-positive cells (CD2, CD3, CD11b, CD14, CD15, CD16, CD19, CD56, CD123, and CD235a) via an ILC2 isolation kit (Miltenyi) (purity > 95%, viability > 99%). The total ILC2 recovery typically ranges from 1 to 5 × 10^6^ cells, depending on the donor.

### NK cell isolation and culture

NK cells were enriched from HD PBMCs via negative selection via a RosetteSep Human NK cell kit (StemCell Technologies) and cultured with NK MACS medium (Miltenyi) supplemented with 10% FBS (Gibco), glutamine, penicillin‒streptomycin and IL-2 (25 ng/ml) plus IL-15 (20 ng/ml) (R&D) for 2 weeks.

### Monocyte and macrophage isolation and coculture

Monocytes were isolated from HD and tumor patient PBMCs via positive selection through magnetic beads via a CD14 MicroBeads Human Kit (Miltenyi). Monocytes were cultured in RPMI-1640 medium supplemented with 10% FBS, glutamine, penicillin–streptomycin (complete medium) and M-CSF (100 ng/ml, Miltenyi) in 24-well ultralow attachment surface plates (Corning) for 7 days to induce their differentiation toward MoM. mPE macrophages were isolated from mPE mononuclear cells via CD14 microbeads like monocytes, but were directly cocultured with ILC2s upon isolation. Coculture experiments were performed between ILC2s after 2 weeks in culture and MoM or mPE macro at a ratio of 1:3 for 4 days. To harvest MoM, the supernatant was discarded, and EDTA (10 mM) diluted in PBS was added to the wells for 10 min at 37 °C to allow for cell detachment. The cells were cocultured for 4 days in 24-well ultralow attachment surface plates in complete RPMI-1640 medium supplemented with IL-2 and IL-7 (50 ng/ml each), with or without a transwell (TW) with a 0.4 µm pore size (Sarstedt). Coculture was also performed in the presence of the monoclonal human antibody nivolumab (anti-PD-1; Selleckchem) (0.5 µg/ml) and purified anti-human CD80 (BioLegend) and anti-human CD86 (Thermo Fisher) (5 µg/mL each).

### In vitro coculture assay of ILC2s and THP-1 cells

THP-1 cells were stained with Cell Tracker Green (CMFDA, Invitrogen) according to the manufacturer’s instructions. THP-1 cells were then cocultured with ILC2s or NK cells at different E:T ratios. After 48 h, the cells were washed twice with cold PBS, incubated for 15 min at RT with an anti-Annexin V antibody and 7-AAD in 1x Annexin binding buffer (BD), and then analyzed by flow cytometry. Cell death was calculated as the percentage of Annexin V^ +^ 7-AAD^+^ cells among the Cell Tracker Green^+^ targets.

### FACS sorting

To isolate ILC2s and MoMs (or mPE macro) after coculture, the cells were harvested, washed and resuspended at a final concentration of 1 × 10^6^ cells/mL in complete RPMI-1640 medium in a sterile FACS tube. The two cell types were separated by cell sorting, taking advantage of their different forward scatter (FSC) and side scatter (SSC). The cells were FACS-sorted with a Cytoflex SRT using a 100 μm nozzle and a sheath pressure of 15 PSI under sterile conditions.

### RNA extraction, reverse transcription, and real-time PCR

Total RNA was extracted from ILC2s with an RNeasy Plus Micro Kit (Qiagen). The RNA was purified from 1 to 5 × 10^5^ cells. The concentration and purity of the RNA were determined with a NanoDrop Spectrophotometer (Thermo Fisher). cDNAs were synthesized via a high-capacity cDNA reverse transcription kit (Thermo Fisher Scientific). Quantitative real-time PCR was performed with the QuantStudio 6 Flex PCR and QuantStudio Real-Time PCR Software v1.3 (Applied Biosystems) using the TaqMan Fast Advanced Master Mix (Thermo Fisher Scientific). The following TaqMan gene expression assays were used: *IL13* (Hs_00174379_m1), *IL5* (Hs_00174200_m1), *IL4* (Hs_00174122_m1), *PDCD1* (Hs01550088_m1), *CTLA4* (Hs00175480_m1), *GAPDH* (Hs00266705_g1) and *18S* (Hs_99999901_s1) conjugated with the fluorochrome FAM (Thermo Fisher). The target gene expression was normalized against the expression of the housekeeping genes *18S* or *GAPDH* and was measured via the threshold cycle (Ct). The fold change was calculated as 2^−ΔΔCt^, where ΔΔCt is the difference between the Ct of the sample and the Ct of the calibrator.

### Bulk RNA sequencing and bioinformatic analysis

For RNA sequencing of ILC2s sorted from coculture with MoMs and ILC2s cultured alone, 3 biological replicates were generated for each condition. The DNA libraries were generated with the TruSeq stranded mRNA prep kit (Illumina, San Diego, California), and RNA sequencing was performed with a NextSeq 500 (Illumina; paired-end reads 2 × 75 cycles with 30 M reads per sample). The fastq files were assessed with the fastqc program, and cutadapt was used to remove the adapter sequence (if present) and the very short reads (read length <20). The samples were mapped to the reference *Homo sapiens* genome (HG38) via the bioinformatics tool STAR [47], and the number of reads mapped to each gene was determined via featureCounts [48]. Normalization and differential analysis were performed with DESeq2 [49]. Differentially expressed genes between ILC2s sorted from coculture and ILC2s cultured alone were then identified from the normalized gene-level counts via the following cutoff values: fold change of 2 and adjusted *P* value ≤ 0.05. PANTHER was utilized to identify pathways enriched with the differentially expressed genes. Genomix4Life (Salerno, Italy) processed the RNA samples and performed differential expression bioinformatics analyses.

### scRNA-seq data analysis

The raw scRNA-seq data of peripheral blood and pleural effusion samples from GSE185058 [24] were processed via Cell Ranger software (10X Genomics). After filtering for low-quality cells and genes with low expression, feature counts were normalized using log1p with a scale factor of 10,000. After performing PCA using highly variable genes, the first 20 principal components were used for dimensionality reduction by t-distributed stochastic neighbor embedding, and graph-based clustering was subsequently performed.

### Soluble cytokines and immune checkpoint ligands analyses

The cytokines and immune checkpoint ligands in the supernatants, plasma and mPEs were detected with different panels of a legendplex multianalyte flow assay kit (BioLegend) according to the manufacturer’s instructions. LEGENDplex HU immune checkpoint panel 1-TC (12-plex) was used to analyze B7.2 (CD86), TGF-β, PD-L1 and PD-L2 expression. A LEGENDplex HU cytokine panel (12-plex) was used to analyze IL-5, IL-13, IL-4, IL-10, IL-6, IL-2 and TNF. LEGENDplex Human Cytokine Panel 2 (13-plex) to analyze IL-1β, GMCSF, TSLP and IL-33. The PL and mPE samples were processed and diluted in the same way prior to analysis. In particular, PL and mPE samples were used neat for analysis with Th cytokine panel and were diluted 1:2 for the Human Cytokine Panel 2 and 1:20 for the Immune checkpoint Panel 1. The data were analyzed with Biolegend Legendplex software.

### Plate-based ligand binding assay to study PD-1 and CTLA-4 function

A flat bottom high-binding 96-well plate (Corning) was incubated overnight at 4 °C with chimeric proteins composed of a recombinant human ligand fused with a linker to human IgG1 Fc (10 µg/ml). The recombinant human ligands used were PD-L1, PD-L2, CD80 and CD86 (Biotechne) (10 µg/ml each). Human IgG1 was used as a negative control, and human serum albumin (HSA) was used at the same concentration as a further negative control for a generic protein. ILC2s were starved for 72 h in complete RPMI-1640 medium without cytokines, after which they were resuspended in complete RPMI-1640 medium supplemented with IL-2 (10 ng/ml) at a final concentration of 5 × 10^4^ cells/well and seeded in precoated plates. The supernatants were collected 24 h post-stimulation to analyze cytokine production.

### Cell stimulation

For PMA/ionomycin stimulation, ILC2s were transferred to 1.5 mL Eppendorf tubes, resuspended in RPMI-1640 complete medium supplemented with phorbol 12–myristate 13–acetate (PMA 10 ng/ml), ionomycin (1 µg/ml), Golgi Stop and Golgi plug, and incubated for 1 hour at 37 °C. The cells were washed and stained as described previously.

For IL-25 and IL-33 stimulation, ILC2s that were sorted from coculture with MoMs and ILC2s that were cultured alone were resuspended in RPMI-1640 complete medium supplemented with IL-25 and IL-33 (50 ng/ml, Miltenyi) and seeded in a 96-well U-bottom plate at a final concentration of 5 × 10^4^ cells/well. The supernatants were collected 3 days post-stimulation to analyze cytokine production.

For LPS stimulation, MoMs that were sorted from coculture with ILC2s and MoMs that were cultured alone were resuspended in RPMI-1640 complete medium supplemented with LPS (100 ng/ml, InvivoGen) and seeded in a 96-well U-bottom plate at a final concentration of 5 × 10^4^ cells/well. The supernatants were collected 20 h post-stimulation to analyze cytokine production.

### Seahorse extracellular flux analysis

Seahorse experiments were performed on ILC2s sorted from cocultures with MoM or ILC2s cultured alone via an XFp T-cell metabolic profiling kit (Seahorse Bioscience). The OCR was measured with an XF Hs Mini Analyzer (Seahorse Bioscience). Briefly, the cells were plated on Agilent Seahorse XF HS PDL miniplates (30,000 cells/well), equilibrated for 1 hour at 37 °C, and assayed for their OCR (pmol/min) under basal conditions and after the addition of oligomycin A (1.5 μM), Bam 15 (2.5 μM), and antimycin A/rotenone (0.5 μM/0.5 μM). All of the Seahorse experiments were performed using the manufacturer’s recommended media (pH 7.4), without phenol red, to standardize the pH conditions of all the samples.

### Statistical analysis

Normality was tested with the Shapiro‒Wilk test to determine whether it was more correct to use a parametric or nonparametric test. Unpaired two-tailed Student’s *t* tests (or paired Student’s *t* tests when comparing data between ILC2s from the same donor) were used if the data followed a Gaussian distribution with similar variances; otherwise, Mann‒Whitney U tests (or Wilcoxon rank sum tests for paired data) were performed. A ratio t test was used to compare the raw values between groups in the experiments where the results are shown as the fold change. For multigroup comparisons, ordinary one-way ANOVA for normally distributed data and the Kruskal‒Wallis test for nonnormally distributed data were used. Differences were considered significant at *P* < 0.05.

## Supplementary information

Supplementary Figures

## Data Availability

The data that support the findings of this study are available from the corresponding author upon reasonable request. The bulk RNA sequencing data have been deposited in the Gene Expression Omnibus (GEO) database under accession code GSE307850.

## References

[1] Vivier E, Artis D, Colonna M, Diefenbach A, Di Santo JP, Eberl G, et al. Innate lymphoid cells: 10 years on. Cell. 2018;174:1054–66. 30142344 10.1016/j.cell.2018.07.017

[2] Bie Q, Zhang P, Su Z, Zheng D, Ying X, Wu Y, et al. Polarization of ILC2s in peripheral blood might contribute to immunosuppressive microenvironment in patients with gastric cancer. J Immunol Res. 2014;2014:923135. 24741632 10.1155/2014/923135 PMC3987940

[3] Salimi M, Wang R, Yao X, Li X, Wang X, Hu Y, et al. Activated innate lymphoid cell populations accumulate in human tumor tissues. BMC Cancer. 2018;18:341. 10.1186/s12885-018-4262-4 PMC5870240 29587679

[4] Chevalier MF, Trabanelli S, Racle J, Salomé B, Cesson V, Gharbi D, et al. ILC2-modulated T-cell-to-MDSC balance is associated with bladder cancer recurrence. J Clin Invest. 2017;127:2916–29. 28650339 10.1172/JCI89717 PMC5531411

[5] Trabanelli S, Chevalier MF, Martinez-Usatorre A, Gomez-Cadena A, Salomé B, Lecciso M, et al. Tumor-derived PGD2 and NKp30-B7H6 engagement drives an immunosuppressive ILC2-MDSC axis. Nat Commun. 2017;8:1–14. 28928446 10.1038/s41467-017-00678-2 PMC5605498

[6] Moral JA, Leung J, Rojas LA, Ruan J, Zhao J, Sethna Z, et al. ILC2s amplify PD-1 blockade by activating tissue-specific cancer immunity. Nature. 2020;579:130–5. 32076273 10.1038/s41586-020-2015-4 PMC7060130

[7] Jacquelot N, Seillet C, Wang M, Pizzolla A, Liao Y, Hediyeh-Zadeh S, et al. Blockade of the coinhibitory molecule PD-1 unleashes ILC2-dependent antitumor immunity in melanoma. Nat Immunol. 2021;22:851–64. 34099918 10.1038/s41590-021-00943-z PMC7611091

[8] Wherry EJ, Kurachi M. Molecular and cellular insights into T-cell exhaustion. Nat Rev Immunol. 2015;15:486–99. 26205583 10.1038/nri3862 PMC4889009

[9] Burke KP, Chaudhri A, Freeman GJ, Sharpe AH. The B7:CD28 family and friends: unraveling coinhibitory interactions. Immunity. 2024;57:223–44. 38354702 10.1016/j.immuni.2024.01.013 PMC10889489

[10] Helou DG, Shafiei-Jahani P, Hurrell BP, Painter JD, Quach C, Howard E, et al. LAIR-1 acts as an immune checkpoint on activated ILC2s and regulates the induction of airway hyperreactivity. J Allergy Clin Immunol. 2022;149:223–236.e6. 34144112 10.1016/j.jaci.2021.05.042 PMC8674385

[11] Taylor S, Huang Y, Mallett G, Stathopoulou C, Felizardo TC, Sun MA, et al. PD-1 regulates KLRG1+ group 2 innate lymphoid cells. J Exp Med. 2017;214:1663–78. 28490441 10.1084/jem.20161653 PMC5461001

[12] Kunitski M, Eicke N, Huber P, Köhler J, Zeller S, Voigtsberger J, et al. Runx/Cbfβ complexes protect group 2 innate lymphoid cells from exhausted-like hyporesponsiveness during allergic airway inflammation. Nat Commun. 2019;10:1–13. 30683858 10.1038/s41467-019-08365-0 PMC6347616

[13] Heinrich B, Gertz EM, Schäffer AA, Craig A, Ruf B, Subramanyam V, et al. The tumor microenvironment shapes innate lymphoid cells in patients with hepatocellular carcinoma. Gut. 2022;71:1161–75. 34340996 10.1136/gutjnl-2021-325288 PMC8807808

[14] Youngblood B, Oestreich KJ, Ha SJ, Duraiswamy J, Akondy RS, West EE, et al. Chronic virus infection enforces demethylation of the locus that encodes PD-1 in antigen-specific CD8(+) T cells. Immunity. 2011;35:400–12. 21943489 10.1016/j.immuni.2011.06.015 PMC3183460

[15] Butte MJ, Keir ME, Phamduy TB, Sharpe AH, Freeman GJ. Programmed death-1 ligand 1 interacts specifically with the B7-1 costimulatory molecule to inhibit T-cell responses. Immunity. 2007;27:111–22. 17629517 10.1016/j.immuni.2007.05.016 PMC2707944

[16] Latchman Y, Wood CR, Chernova T, Chaudhary D, Borde M, Chernova I, et al. PD-L2 is a second ligand for PD-1 and inhibits T-cell activation. Nat Immunol. 2001;2:261–8. 11224527 10.1038/85330

[17] Yu Y, Tsang JC, Wang C, Clare S, Wang J, Chen X, et al. Single-cell RNA-seq identifies a PD-1hi ILC progenitor and defines its development pathway. Nature. 2016;539:102–6. 27749818 10.1038/nature20105

[18] Helou DG, Shafiei-Jahani P, Lo R, Howard E, Hurrell BP, Galle-Treger L, et al. PD-1 pathway regulates ILC2 metabolism and PD-1 agonist treatment ameliorates airway hyperreactivity. Nat Commun. 2020;11:1–15. 32778730 10.1038/s41467-020-17813-1 PMC7417739

[19] Ebihara T, Taniuchi I. Exhausted-like group 2 innate lymphoid cells in chronic allergic inflammation. Trends Immunol. 2019;40:1095–104. 31735510 10.1016/j.it.2019.10.007

[20] Spits H, Mjösberg J. Heterogeneity of type 2 innate lymphoid cells. Nat Rev Immunol. 2022;22:701–12. 35354980 10.1038/s41577-022-00704-5 PMC8966870

[21] Saranchova I, Han J, Zaman R, Arora H, Huang H, Fenninger F, et al. Type 2 innate lymphocytes actuate immunity against tumors and limit cancer metastasis. Sci Rep. 2018;8:1–17. 29440650 10.1038/s41598-018-20608-6 PMC5811448

[22] Bahhar I, Eş Z, Köse O, Turna A, Günlüoğlu MZ, Çakır A, et al. The IL-25/ILC2 axis promotes lung cancer with a concomitant accumulation of immune-suppressive cells in tumors in humans and mice. Front Immunol. 2023;14:1244437. 37781372 10.3389/fimmu.2023.1244437 PMC10540623

[23] Stathopoulos GT, Kalomenidis I. Malignant pleural effusion: tumor-host interactions unleashed. Am J Respir Crit Care Med. 2012;186:487–92. 22652027 10.1164/rccm.201203-0465PP PMC5650050

[24] Huang ZY, Shao MM, Zhang JC, Yi FS, Du J, Zhou Q, et al. Single-cell analysis of diverse immune phenotypes in malignant pleural effusion. Na Commun. 2021;12:1–12. 10.1038/s41467-021-27026-9 PMC8602344 34795282

[25] Shen C, Liu C, Zhang Z, Ping Y, Shao J, Tian Y, et al. PD-1 affects the immunosuppressive function of group 2 innate lymphoid cells in human non-small cell lung cancer. Front Immunol. 2021;12:680055. 34194433 10.3389/fimmu.2021.680055 PMC8237944

[26] Li Z, Ma R, Tang H, Guo J, Shah Z, Zhang J, et al. Therapeutic application of human type 2 innate lymphoid cells via induction of granzyme B-mediated tumor cell death. Cell. 2024;187:624–641.e23. 38211590 10.1016/j.cell.2023.12.015 PMC11442011

[27] Reid KT, Colpitts SJ, Mathews JA, Santos Carreira A, Murphy JM, Borovsky DT, et al. Cell therapy with human IL-10-producing ILC2s limits xenogeneic graft-versus-host disease by inhibiting pathogenic T-cell responses. Cell Rep. 2025;44:115102. 10.1016/j.celrep.2024.115102 39721022

[28] Lim AI, Menegatti S, Bustamante J, Le Bourhis L, Allez M, Rogge L, et al. IL-12 drives functional plasticity of human group 2 innate lymphoid cells. J Exp Med. 2016;213:569–83. 26976630 10.1084/jem.20151750 PMC4821648

[29] Ogasawara N, Poposki JA, Klingler AI, Tan BK, Weibman AR, Hulse KE, et al. IL-10, TGF-β, and glucocorticoid prevent the production of type 2 cytokines in human group 2 innate lymphoid cells. J Allergy Clin Immunol. 2018;141:1147–1151.e8. 29074458 10.1016/j.jaci.2017.09.025 PMC5844803

[30] Follows ER, McPheat JC, Minshull C, Moore NC, Pauptit RA, Rowsell S, et al. Study of the interaction of the medium chain mu 2 subunit of the clathrin-associated adapter protein complex 2 with cytotoxic T-lymphocyte antigen 4 and CD28. Biochem J. 2001;359:427–34. 11583591 10.1042/0264-6021:3590427 PMC1222163

[31] Nirschl CJ, Drake CG. Molecular pathways: coexpression of immune checkpoint molecules: Signaling pathways and implications for cancer immunotherapy. Clin Cancer Res. 2013;19:4917–24. 23868869 10.1158/1078-0432.CCR-12-1972 PMC4005613

[32] Scharping NE, Menk AV, Moreci RS, Whetstone RD, Dadey RE, Watkins SC, et al. The tumor microenvironment represses T-cell mitochondrial biogenesis to drive intratumoral T-cell metabolic insufficiency and dysfunction. Immunity. 2016;45:374–88. 27496732 10.1016/j.immuni.2016.07.009 PMC5207350

[33] Yue J, Guo H, Xu P, Ma J, Shi W, Wu Y. Combination of IL-33 with PD-1 blockade augment mILC2s-mediated anti-tumor immunity. Cancer Immunol Immunother. 2024;73:65. 38430390 10.1007/s00262-023-03580-7 PMC10908611

[34] van der Ploeg EK, Golebsky K, van Nimwegen M, Fergusson JR, Heesters BA, Martinez-Gonzalez I, et al. Steroid-resistant human inflammatory ILC2s are marked by CD45RO and elevated in type 2 respiratory diseases. Sci Immunol. 2021;6:eabd3489. 10.1126/sciimmunol.abd3489 33514640

[35] Chikuma S, Imboden JB, Bluestone JA. Negative regulation of T-cell receptor-lipid raft interaction by cytotoxic T lymphocyte-associated antigen 4. J Exp Med. 2003;197:129–35. 12515820 10.1084/jem.20021646 PMC2193802

[36] Pentcheva-Hoang T, Chen L, Pardoll DM, Allison JP. Programmed death-1 concentration at the immunological synapse is determined by ligand affinity and availability. Proc Natl Acad Sci USA. 2007;104:17765–70. 17968013 10.1073/pnas.0708767104 PMC2077030

[37] Niu H, Zhang H, Wang D, Zhao L, Zhang Y, Zhou W, et al. LKB1 prevents ILC2 exhaustion to enhance antitumor immunity. Cell Rep. 2024;43:113579. 38670109 10.1016/j.celrep.2023.113579

[38] Egen JG, Allison JP. Cytotoxic T lymphocyte antigen-4 accumulation in the immunological synapse is regulated by TCR signal strength. Immunity. 2002;16:23–35. 11825563 10.1016/s1074-7613(01)00259-x

[39] Rusilowicz-Jones EV, Urbé S, Clague MJ. Protein degradation on the global scale. Mol Cell. 2022;82:1414–23. 35305310 10.1016/j.molcel.2022.02.027

[40] Valk E, Rudd CE, Schneider H. CTLA-4 trafficking and surface expression. Trends Immunol. 2008;29:272–9. 18468488 10.1016/j.it.2008.02.011 PMC4186961

[41] Tey PY, Dufner A, Knobeloch K, Pruneda JN, Clague MJ, Urbé S, Rapid turnover of CTLA4 is associated with a complex architecture of reversible ubiquitylation. J Cell Biol. 2025;224:e202312141. 10.1083/jcb.202312141 PMC11486831 39404738

[42] Lo JW, Schroeder JH, Roberts LB, Mohamed R, Cozzetto D, Beattie G, et al. CTLA-4 expressing innate lymphoid cells modulate mucosal homeostasis in a microbiota dependent manner. Nat Commun. 2024;15:9520. 39496592 10.1038/s41467-024-51719-6 PMC11535242

[43] Ahmed A, Joseph AM, Zhou J, Horn V, Uddin J, Lyu M, et al. CTLA-4-expressing ILC3s restrain interleukin-23-mediated inflammation. Nature. 2024;630:976–83. 38867048 10.1038/s41586-024-07537-3 PMC11298788

[44] Rudd CE. The reverse stop-signal model for CTLA4 function. Nat Rev Immunol. 2008;8:153–60. 18219311 10.1038/nri2253

[45] Bengsch B, Johnson AL, Kurachi M, Odorizzi PM, Pauken KE, Attanasio J, et al. Bioenergetic insufficiencies due to metabolic alterations regulated by the inhibitory receptor PD-1 are an early driver of CD8(+) T-cell exhaustion. Immunity. 2016;45:358–73. 27496729 10.1016/j.immuni.2016.07.008 PMC4988919

[46] Van Gassen S, Callebaut B, Van Helden MJ, Lambrecht BN, Demeester P, Dhaene T, et al. FlowSOM: Using self-organizing maps for visualization and interpretation of cytometry data. Cytometry Part. 2015;87:636–45. 10.1002/cyto.a.22625 25573116

[47] Dobin A, Davis CA, Schlesinger F, Drenkow J, Zaleski C, Jha S, et al. STAR: ultrafast universal RNA-seq aligner. Bioinformatics. 2013;29:15–21. 23104886 10.1093/bioinformatics/bts635 PMC3530905

[48] Liao Y, Smyth GK, Shi W. The Subread aligner: fast, accurate and scalable read mapping by seed-and-vote. Nucleic Acids Res. 2013;41:e108–e108. 23558742 10.1093/nar/gkt214 PMC3664803

[49] Love MI, Huber W, Anders S. Moderated estimation of fold change and dispersion for RNA-seq data with DESeq2. Genome Biol 2014;15:550. 10.1186/s13059-014-0550-8 PMC4302049 25516281

