# Supplementary material for: Macrophages regulate PD-1 and CTLA-4 expression on ILC2s and their responsiveness in the tumor microenvironment
Source: Cell Mol Immunol. 2025 Sep 24;22(11):1491–505. doi: 10.1038/s41423-025-01347-x (PMC12575681; doi:10.1038/s41423-025-01347-x)
Supplement: Supplementary file 1 — Supplementary Figures [file 41423_2025_1347_MOESM1_ESM.pdf]

Supplementary Figure 1

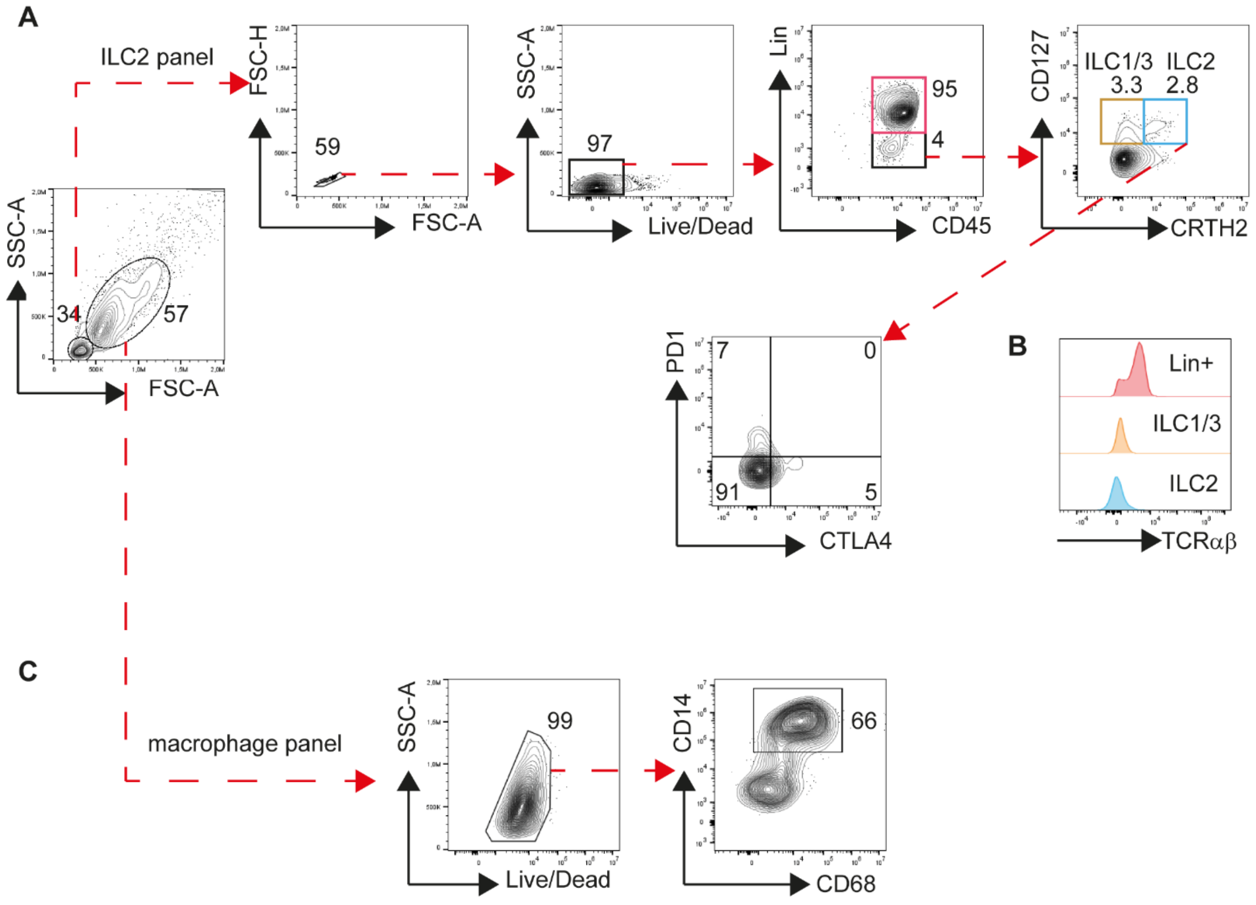

Representative gating strategy for ILC (A) and macrophages (C) analysis in mononuclear cells isolated from mPE. (B) TCRαβ expression by Lin(CD3, CD14, CD19, CD20, CD56, CD16)$^{+}$ cells, ILC1/3 and ILC2 is shown.

Supplementary Figure 2

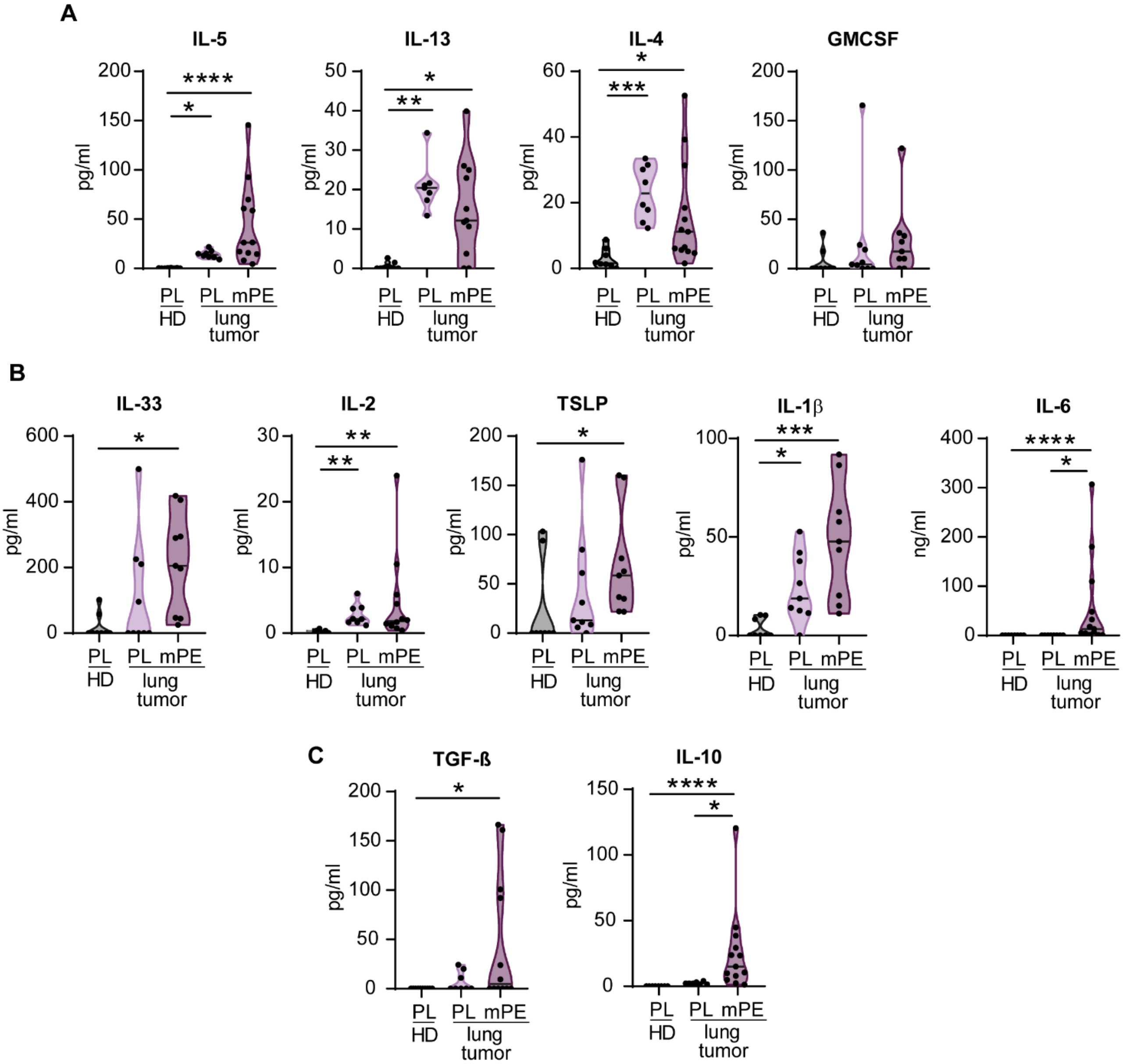

Concentration of cytokines in HD PB, PB and mPE of lung tumor patients. Each dot represents one donor. One-way ANOVA was performed; *p<0.05, **p<0.01, ***p<0.0005, ****p<0.0001 (A-C).

Supplementary Figure 3

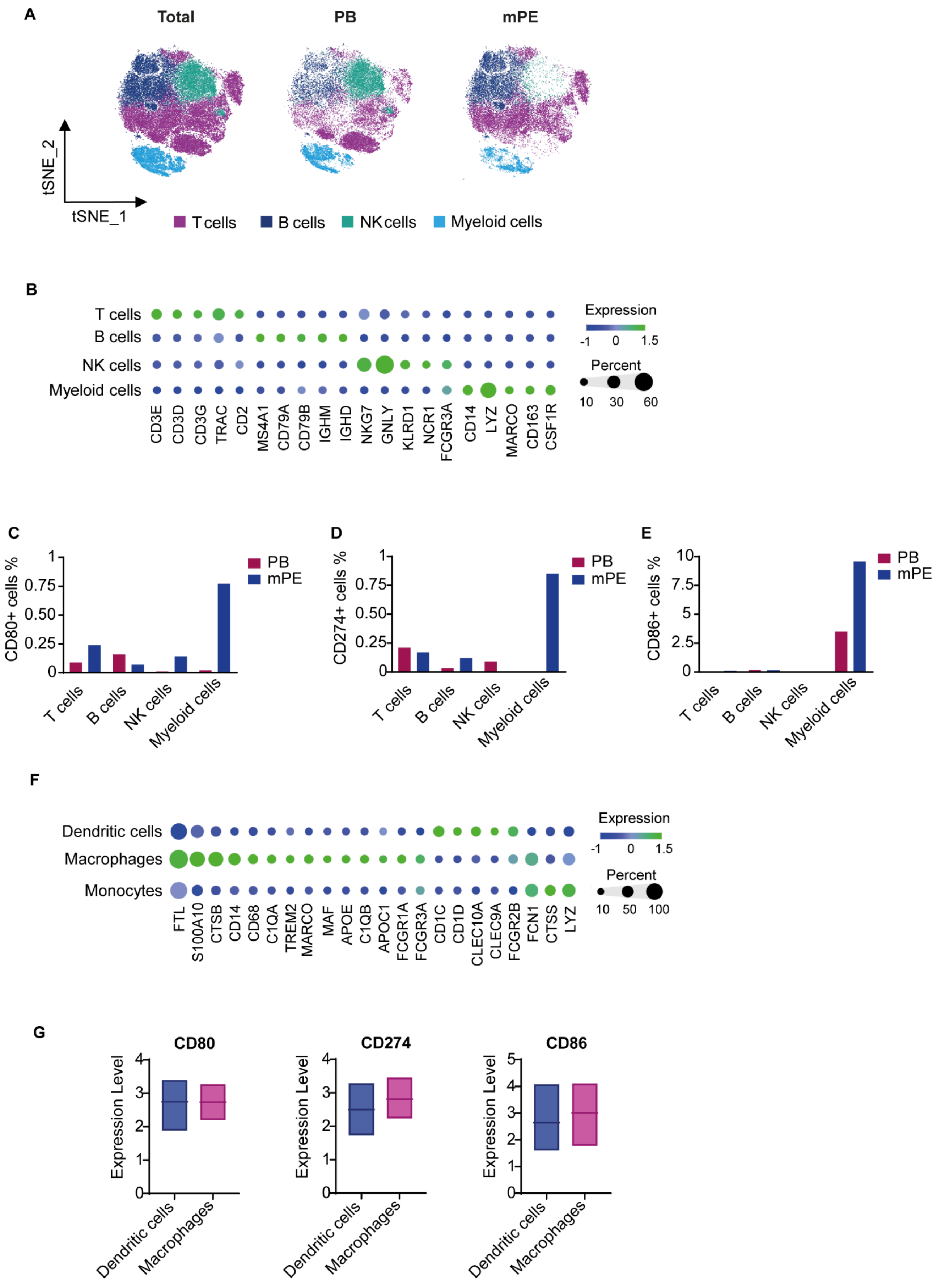

(A) tSNE plots of total, peripheral blood (PB) and pleural effusion (mPE) samples from scRNAseq data (Huang et al., 2021), color-coded by cell type. (B) Dot plot of average expression of canonical marker genes for the cell types defined in A. (C-E) Percentage of cells expressing CD80, CD86 and CD274 genes in PB and mPE samples. (F) Dot plot of average expression of canonical marker genes for the myeloid cell subsets defined in Figure 2B. (G) CD80, CD86 and CD274 gene expression in dendritic cells and macrophages from mPE samples. PB, n = 5 samples, mPE, n = 5 samples

Supplementary Figure 4

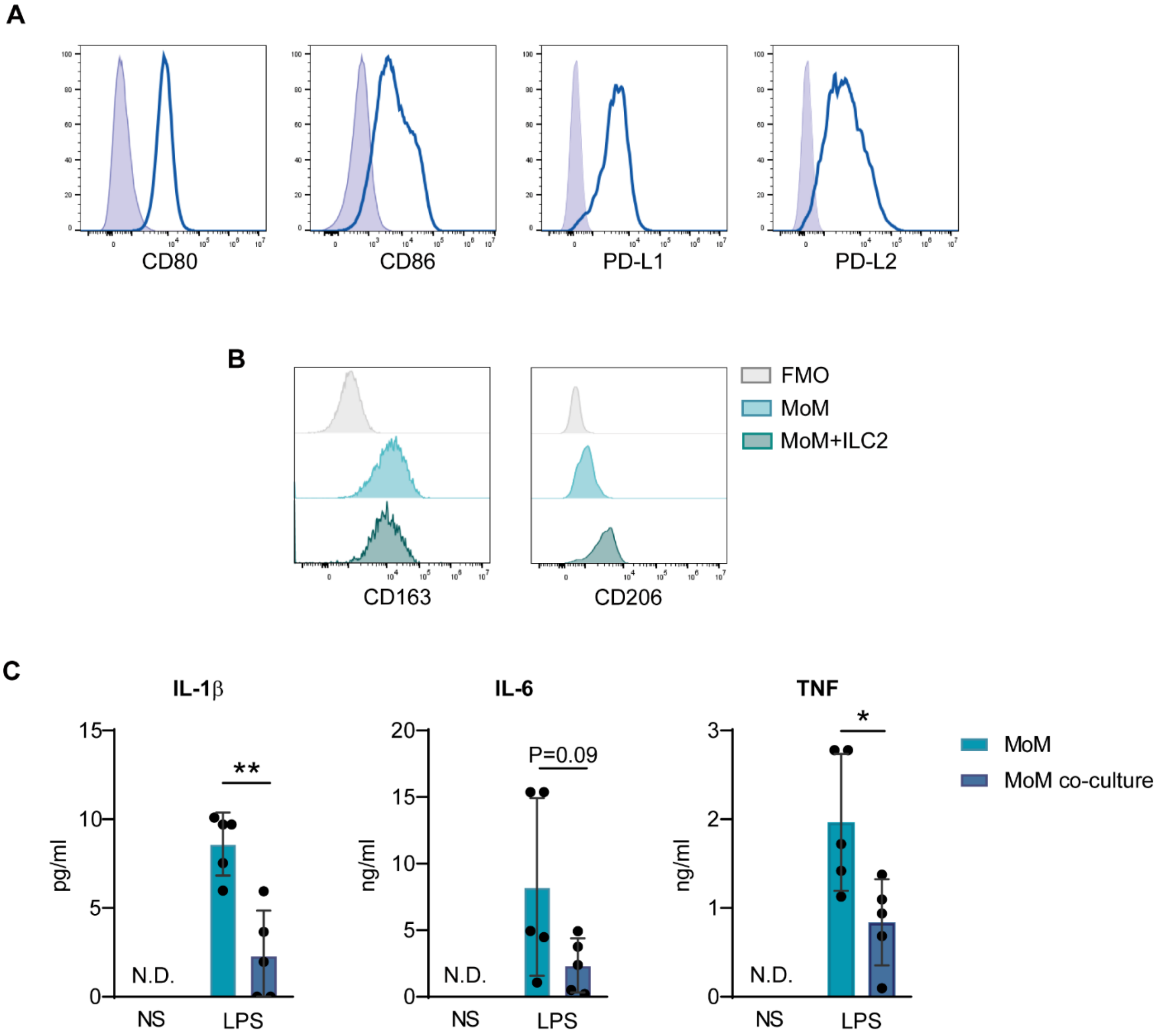

CTLA-4 ligands (CD80, CD86) and PD-1 ligands (PD-L1, PD-L2) expression was analyzed on MoM generated from HD PBMCs. FACS histograms are shown for one representative donor. FMO is shown as a negative control (A). CD163 and CD206 expression on MoM cultured alone or with ILC2, FMO is shown as a negative control. FACS histograms are shown for one representative donor (B). The histograms show the concentration of IL-1β, IL-6 and TNF released in the supernatant by MoM in response to stimulation with LPS. The not stimulated (NS) cells did not release cytokines. N.D.= not detected. Cytokine concentrations are expressed as mean +/- SD of 5 independent experiments. T-test was performed between stimulated samples; *$p<0.05$, **$p<0.01$ (C).

Supplementary Figure 5

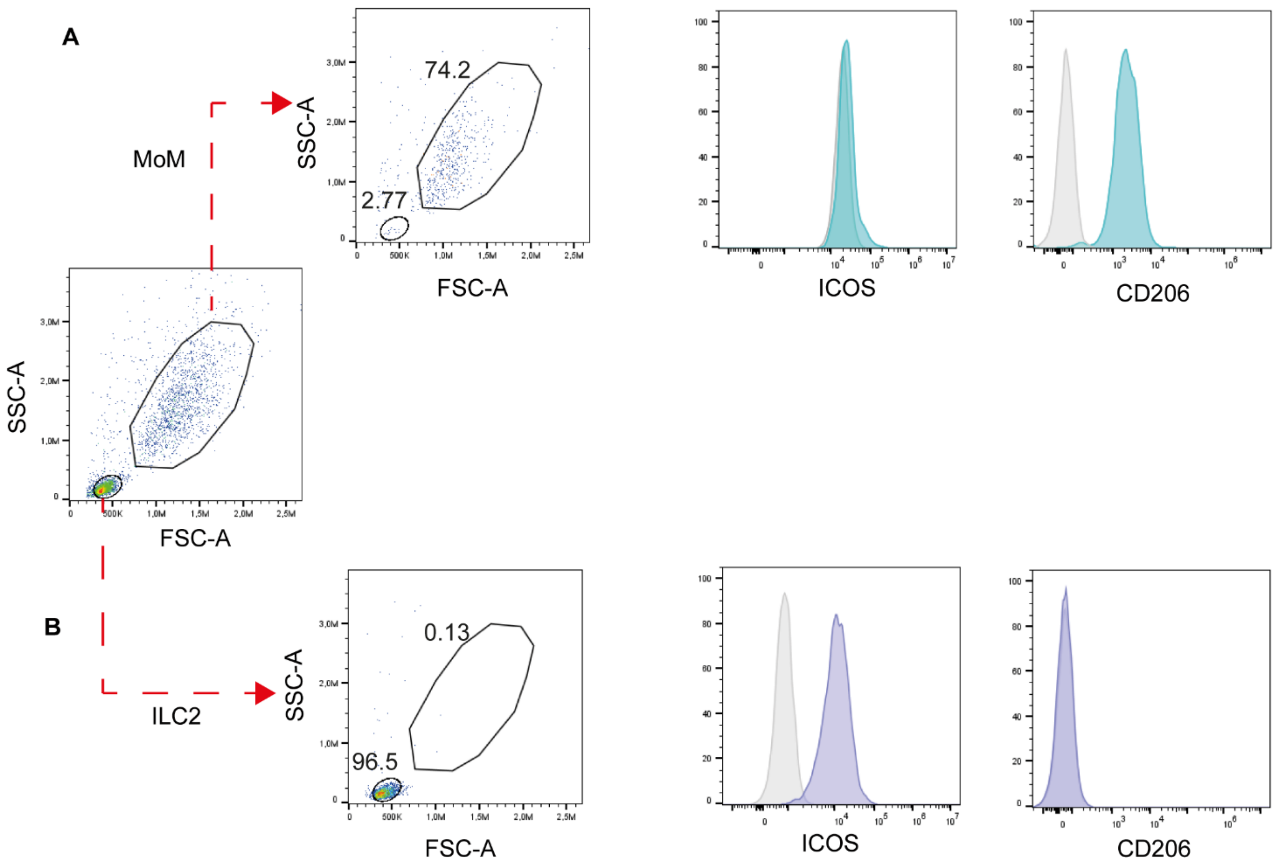

Pre- and post-sort analysis of side/forward scatter profiles of MoM (A) and ILC2 (B) after co-culture. Post-sort purity was verified by analyzing on the flow sorted populations the expression of ICOS and CD206. Representative flow cytometry histograms are shown. FMO (grey histogram) is shown as a negative control.

Supplementary Figure 6

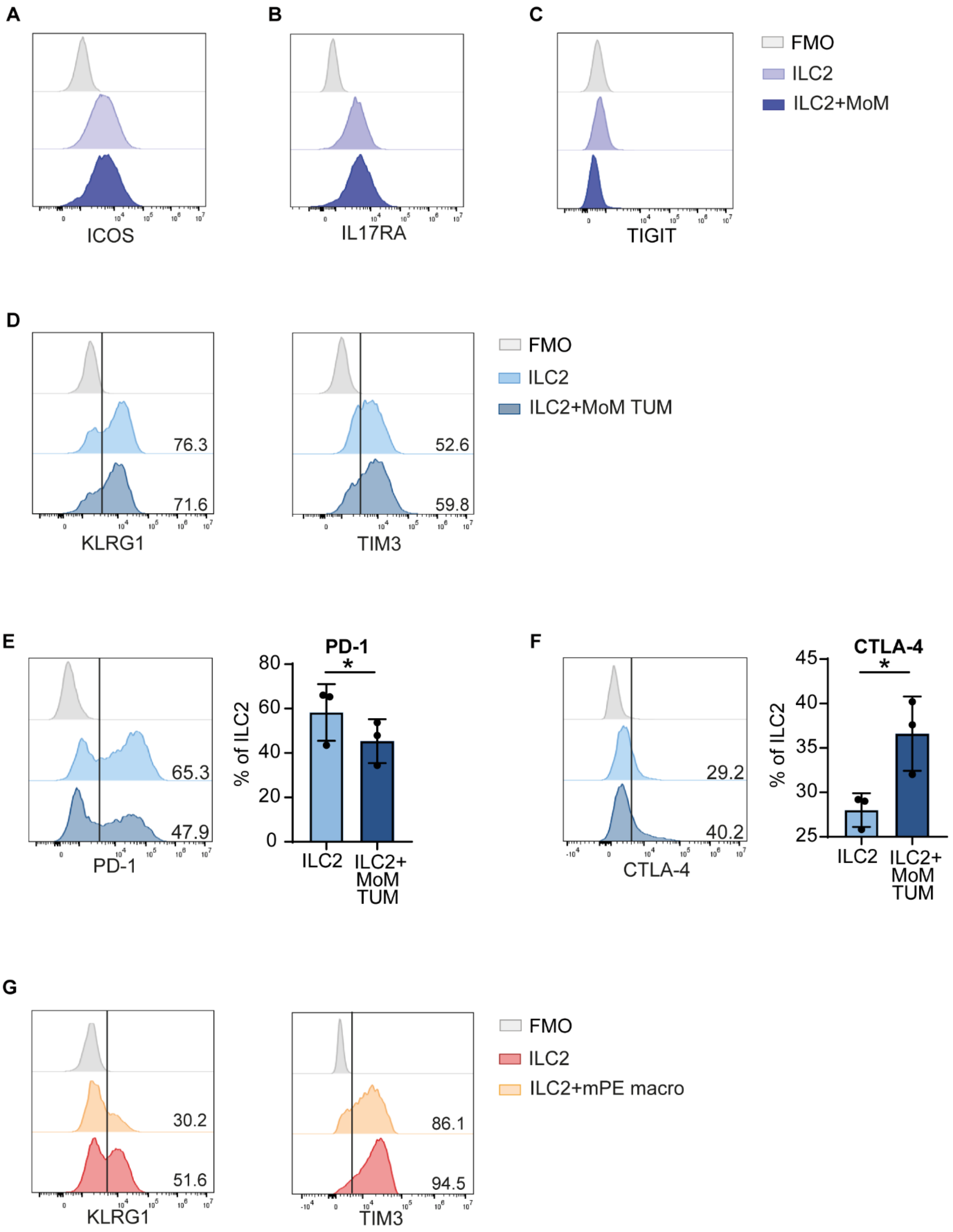

Representative flow cytometry histograms of the expression of the indicated receptors on ILC2 cultured alone or co-cultured with MoM (A-C), MoM Tum (D-F) and mPE macro (G). FMO (grey histogram) is shown as a negative control. In E and F the mean +/- SD of 3 independent experiments is shown. T-test was performed; *$p<0.05$.

Supplementary Figure 7

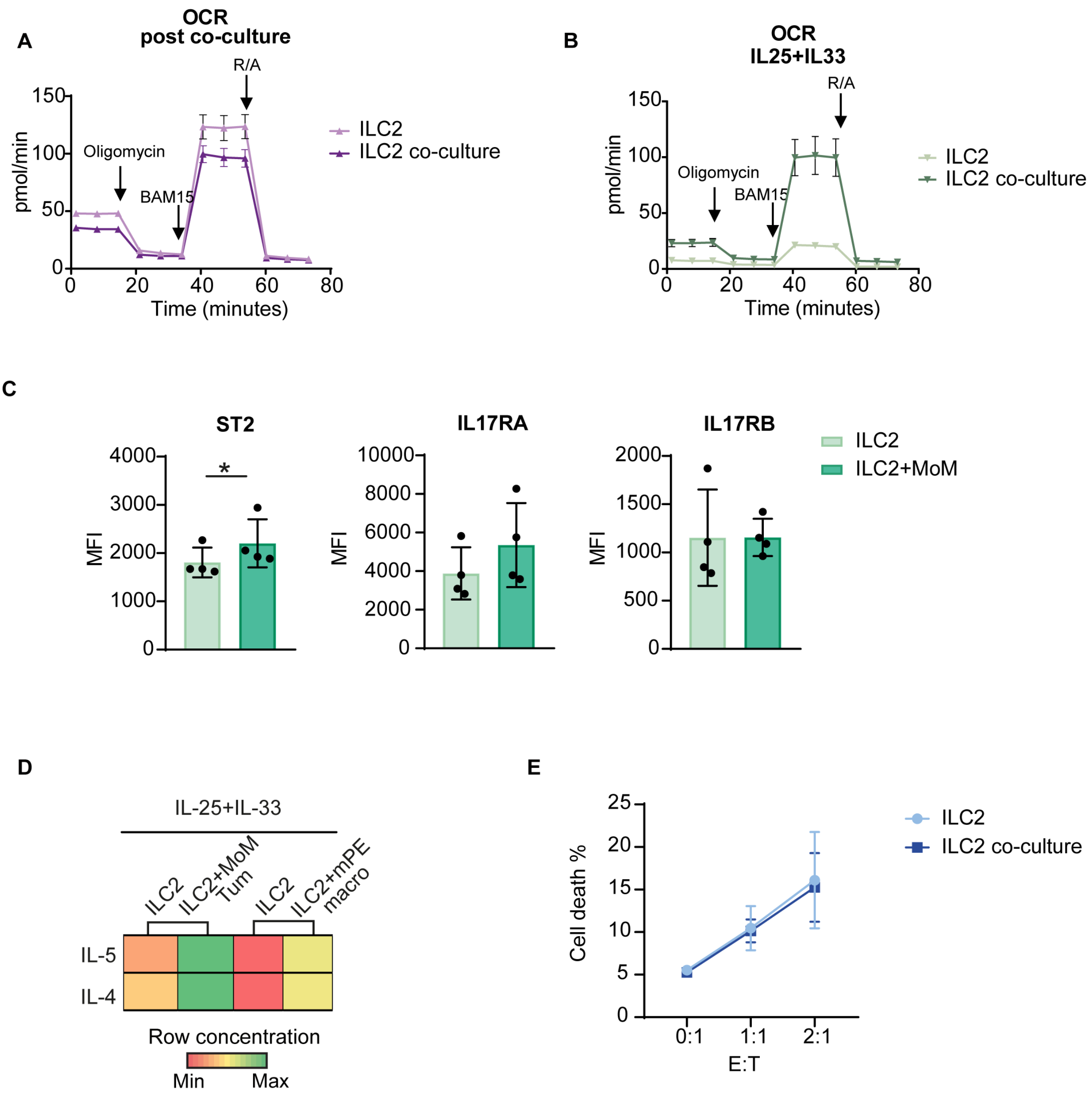

(A-C) ILC2 were cultured alone (ILC2) or co-cultured with MoM (ILC2 co-culture) and then stimulated with IL25+IL-33 for 3 days. Seahorse analysis of the oxygen consumption rate (OCR) was performed immediately (A) and after stimulation with IL-25 and IL-33 (B). (C) ILC2 expression of ST2, IL17RA and IL17RB after stimulation with IL-25 and IL-33. Data are shown as mean +/- SD of 4 independent experiments. T-test was performed (*$p<0.05$). (D) Concentrations in the supernatant of the cytokines released by ILC2 stimulated with IL-25 and IL-33 for 3 days, after co-culture with MoM Tum and mPE macro. Mean data of 2 independent experiments for each co-culture condition are shown. (E) Percentage of dead cells among THP-1 target cells (identified as Annexin V^+/7-AAD^+), after 48h co-culture with ILC2 (previously cultured alone or co-cultured with MoM) at the indicated effector:target (E:T) ratios. Mean +/- SD of 2 independent experiments is shown.
